# Institutional Quality in Green and Digital Transition of EU Regions – A Recovery and Resilience Analysis

**DOI:** 10.1002/gch2.202400031

**Published:** 2024-08-20

**Authors:** Alexandru Bănică, Ramona Ţigănaşu, Peter Nijkamp, Karima Kourtit

**Affiliations:** ^1^ Faculty of Geography “Alexandru Ioan Cuza” University of Iasi, Romania, Center for Geographic Research Romanian Academy Iasi Branch 700506 Romania; ^2^ Centre for European Studies “Alexandru Ioan Cuza” University of Iasi Iasi 700507 Romania; ^3^ Open University Heerlen 6401 the Netherlands

**Keywords:** green and digital transition, institutional quality, National Recovery and Resilience Plans, regional disparities, strategic priorities, sustainable development

## Abstract

This paper assesses the National Recovery and Resilience Plans (NRRPs) of EU member states and regions to uncover commonalities and differences between green and digital transitions, focusing on the role of institutions, among additional socio‐economic drivers, in modeling them. To that end, relevant indicators have been assembled, and several econometric models have been developed and tested to evaluate institutional performance in relation to green and digital transformations. The study reveals discrepancies in the two explored transition fields and highlights the power of institutional factors in boosting them. Specifically, the findings demonstrate that the green transition in EU regions is positively associated with variables such as life expectancy, institutional quality, tertiary education attainment, and small and medium enterprises (SMEs) with innovative activities, while the fruits of digitalization are mainly allied to population with higher studies, core creative class employment, accountability of institutions, and innovative SMEs. These insights offer valuable guidance for decision‐makers to draw lessons from high‐performing or successful regions and strategically assign resources. This includes paying attention to regional financial allocations and their alignment with territorial planning and long‐term policies.

## Graphical abstract

The study uncovers commonalities and differences between the “twin transitions” at national and regional levels in the EU, focusing on the role of institutions, among other socio‐economic drivers, in shaping them. It concludes that green and digital transitions are robustly influenced by human capital, innovative activities, and institutional accountability.

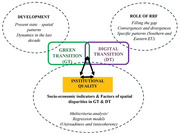

## 1 Introduction

The alignment of environmental goals within the European Green Deal (EGD) with economic growth objectives has been widely discussed in recent years. Moreover, the integration of new digital technologies to facilitate these objectives has been a prominent topic. Although official strategies and policy documents may suggest a clear roadmap for EU member states, the practical realities are far more intricate. Each nation must consider its individual capacities and capabilities, recognizing that they do not commence growth processes from the same starting point. Various factors account for these disparities. Furthermore, environmental challenges cannot always be entirely controlled, highlighting the governments' inability to ensure complete predictability, irrespective of technological advancement. Consequently, while technology can streamline the ecological transition, it does not necessarily mitigate natural hazards. Nevertheless, it is important to acknowledge the impact of technological determinants on economic and social phenomena, which often overshadow environmental considerations.^[^ ^1^ ^]^

Individuals bear a great responsibility in environmental matters, and digitalization can play a decisive role in promoting ecological behaviors (of people, industry, and society). However, the adoption of modern technologies carries costs, creating gaps among countries. An approach centered primarily on the digital dimension can unintentionally disrupt factors shaping the green transition. Achieving a perfect balance between the economy and the environment remains elusive, primarily due to regional disparities. Some regions possess favorable factors for economic clustering, often linked to environmental damage, while others, isolated and eco‐friendly, may face challenges appearing from poor interconnectivity. This raises the question of the type of governance that can truly support sustainability and digitalization purposes while innovating and improving well‐being effectively.^[^ ^2^ ^]^ Ansell et al.^[^ ^3^ ^]^ argue that robust governance is often advocated, in which change and stability, though distinct concepts coexist symbiotically, to the detriment of bureaucratic governance, which subverts market mechanisms or even network governance, with its flexibility and focus on problem‐solving, that may fall short. Such robust governance should be complemented by institutional responsiveness, aligning development strategies with a state's capabilities, setting realistic objectives, and engaging responsibly in their fulfilment.^[^ ^4^ ^]^

In recent years, the concept of a “regenerative society”, focusing on minimizing CO2 emissions and efficient resource utilization, has gained scientific traction.^[^ ^5^, ^6^, ^7^ ^]^ Given the digital era we now inhabit, it is essential to explore whether a successful transition to a circular economy can occur without appropriate institutional integration or coordination. Consequently, several key questions emerge, which we aim to address in this study:

1. Can high institutional quality simultaneously ensure a cleaner environment and a more significant digitalization absorption?
2. Should the green and digital dimensions be treated as “twins,” or is it more effective to address them separately for optimal results?
3. Do less developed European regions possess the capacity to implement in parallel environmental and digital objectives?

With these questions in mind, our central research objective is to analyze how various socio‐economic factors, along with the role of institutions and governance, influence green and digital transitions in EU regions, particularly in areas receiving substantial funding from the Recovery and Resilience Facility (RRF) program of the EU. We aim to determine whether there is spatial convergence or divergence between the two transitions and the underlying institutional framework.

An initial exploratory literature review on this issue revealed that the combination of the green‐digital‐institutional trinomial is rarely addressed. Most works focus on bilateral relationships, such as between digitalization and sustainability,^[^ ^8^, ^9^, ^10^, ^11^, ^12^, ^13^ ^]^ without considering the broader array of issues, especially at the sub‐national territorial level. This underscores the sensitivity of actions. Furthermore, the influence of institutions on the green component remains inadequately covered, with the literature mainly discussing the capacity of institutions to drive economic growth.^[^ ^14^, ^15^, ^16^, ^17^ ^]^ Therefore, our original approach seeks to comprehensively study the factors explaining green and digital transitions within EU regions, placing institutions at the center of the analysis, to determine whether they facilitate or hinder these transitions, either individually or collectively. Regional disparities within the EU can erode trust in institutions,^[^ ^18^ ^]^ but recognizing institutional dysfunction allows for corrective action,^[^ ^19^ ^]^ especially in the current context, where information technology and artificial intelligence systems can support decision‐makers to promote regional development.^[^ ^20^, ^21^ ^]^ This calls for an integrative perspective.

**Figure 1:**
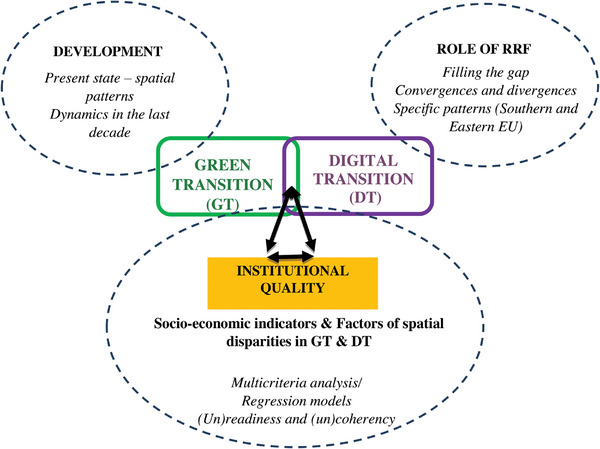
Conceptual framework.

The conceptual framework, as depicted in **Figure** **1**, explores the interaction between institutional quality at the national or regional level and the elements of the “twin” transitions, as presented below.

Such a strategic approach is essential to strengthen the resilience of territories, as institutions are considered the catalysts of smart activities, promoting the efficient diffusion of innovation,^[^ ^22^, ^23^ ^]^ including digital and green innovation. High‐quality local institutions can enhance human capital, productivity, and innovative capacity.^[^ ^24^ ^]^ Therefore, institutional quality must be recognized as a significant factor in addressing green innovation and sustainability issues.^[^ ^25^ ^]^ This is the subject matter of the present study.

By referring to these, our paper not only seeks to explore the link between digital and green components, but also to fill the gap in the literature, enclosing elements that define the economic and institutional constituents, in order to emphasize how they interact in the evolution of the twin transition at the regional level, which is far less exploited. A study applied to regions can more faithfully indicate the differences within the countries, but nevertheless, by incorporating a large number of very miscellaneous regions from an economic and institutional standpoint, it is more demanding to propose measures aimed at the recovery of each one. Considering this limit, we framed the regions using cardinal points (Southern, Eastern, Northern, and Western), offering an image in relation to the center‐periphery distinction.

To address these challenges, our article is structured as follows: the next section provides an overview of the state of the art concerning the three research pillars (green, digital, and institutions). In subsequent sections, we focus on the spatial patterns of green and digital transitions and the factors generating disparities. The results section presents the dynamics of green transition and digitalization within the EU, similarities and differences in NRRPs for planning digital and green transitions, and the main drivers in these processes. The paper continues with discussions on the results and, ultimately, the conclusions.

### 1.1 Literature Review

Strengthening economies after significant shocks is a complex process influenced by a country's developmental maturity, technological advancement, and commitment to achieving a certain level of performance. The EU's recovery and resilience tools, which target both private and public actors in member states, aim to facilitate this adjustment process in the aftermath of the COVID‐19 perturbation. The NRRPs are expected to bring changes to how politics is conducted, potentially mitigating controversies from Eurosceptics.^[^ ^26^ ^]^ Even if NRRPs are not permanent, they provide an opportunity for countries to revive their economies, particularly in the face of concurrent crises such as the COVID‐19 pandemic and the conflict in Ukraine. In response to these challenges, the European Parliament introduced the REPowerEU Plan in February 2023, with its main objectives focusing on accelerating the transition to clean energy and enhancing resilience.^[^ ^27^, ^28^ ^]^ These supranational financing instruments may serve as indicators for the future financing direction of the EU, emphasizing the importance of achieving effective results.^[^ ^29^ ^]^ However, there are challenges related to financing ecological transformation, especially in less prosperous countries, where loan repayment capacity is low, making the shift to green economies a formidable task.^[^ ^30^ ^]^ This raises the question of how well‐prepared lagging regions are for digital and green transitions and whether the implementation of NRRPs will reduce or exacerbate regional discrepancies.

While the full results of the transition from the COVID‐19 pandemic to recovery are not yet evident, it is essential to assess the weaknesses and needs within regions, aiming to address disparities and enhance strategic planning, institutional adaptability, mutuality, accountability, innovation, and positive adaptation. Crises can serve as turning points for countries, providing opportunities for reset and revitalization toward more sustainable paths, especially when guided by effective governance.^[^ ^31^, ^32^, ^33^, ^34^, ^35^, ^36^ ^]^

Adapted to current realities, environmental sustainability depends, plainly, on the ability of institutions to integrate digital technologies in multiple sectors and to map out alternatives to replace systems and mechanisms proven to be highly polluting. Modernizing production equipment, as well as supporting small and medium‐sized enterprises (SMEs) in innovative and digital activities can be a key step in mitigating carbon emissions.^[^ ^37^ ^]^ Moreover, the financing of green energy remains a rather pressing problem for regions with a low GDP or modest investment levels. A series of government projects, in line with EGD directives, can come in support of entrepreneurs and households by offering fiscal facilities in this regard.^[^ ^38^, ^39^ ^]^ Apart from the financial aspects, the environmental regulations have to be well established, and the interest in ecological technology should be given priority. However, looking at the EGD, it is found that it means much more than actions for the green objectives and specific policies, related to each state, but rather this initiative aims to dynamize the constructs of European economic model, centered on a future‐oriented environment.^[^ ^40^ ^]^ On this path, sustainable energy is a key concept introduced in the Sustainable Development Goals (SDGs), and its connection with the environment is indisputable. Green technological innovation can have a positive impact on low‐carbon transformation by improving energy efficiency, as suggested by several studies,^[^ ^41^, ^42^, ^43^, ^44^ ^]^ but the diffusion of its effects is extremely different within the EU regions, indicating a center‐periphery gap, which is deepened by economic factors. The European energy markets suffered changes in the context of the war in Ukraine, the EU being forced to adjust its policies; however, regardless of the geopolitical environment, the transition toward clean energy is vital and calls for green technological leadership.^[^ ^45^ ^]^

The NRRPs provide an opportunity for states to drive digital transformation, with its connection to overall economic growth and territorial planning being emphasized in the literature.^[^ ^46^, ^47^, ^48^ ^]^ Regarding the achievement of the SDGs, Palos‐Sánchez et al.^[^ ^49^ ^]^ analyzed how digitalization contributes to this and emphasized the importance of cybersecurity for ensuring the effectiveness of the measures applied, while Yin et al.^[^ ^50^ ^]^ highlighted the contribution of digitalization in narrowing the gender gap. The interconnectivity of people and places is crucial for greater resilience,^[^ ^51^ ^]^ and the environmental impact of digitalization is uneven at urban and regional levels, influenced by factors such as industrialization, landforms, economic conditions, and more, with digitalization's positive effects felt more in areas where polluting activities are relocated.^[^ ^52^ ^]^ Disparities in digitalization, including access to new technologies, skills, and connectivity, are influenced by various socio‐economic indicators.^[^ ^53^ ^]^ A fundamental condition in reducing disparities is the “twin” transition, as articulated by Bianchini et al.,^[^ ^54^ ^]^ encompassing the Green Transition (GT) with a minimum allocation of 37% of the funds and the Digital Transition (DT) with a minimum of 20%. While all EU countries acknowledge the necessity of investments in both areas, the implementation and commitment to achieving these goals may vary. The role of institutions in responsibly facilitating both GT and DT and addressing potential conflicts between these two crucial European priorities cannot be overstated. These conflicts may arise during the prioritization process or due to the fact that not all digital solutions have a positive environmental impact.

The relationship between digitalization and sustainability, or “sustainable digitalization,” is widely discussed in the literature, emphasizing the conditionality of this connection.^[^ ^55^ ^]^ “Digital sustainability” involves activities that advance SDGs by creatively deploying smart technologies.^[^ ^56^ ^]^ George et al.^[^ ^57^ ^]^ assess “digital sustainability” as a tool not just to protect the environment but also to become more competitive, innovative, and institutionally performant. The concept of “digitainability” is used to analyze the impact of digitalization on the fulfillment of SDGs, identifying five types of impact: synergy, ambivalent, trade‐off, uncertain, or bidirectional.^[^ ^58^ ^]^ Various studies explore the hypothesis that digitalization enhances environmental performance, green economy, circular economy, and sustainability.^[^ ^59^, ^60^, ^61^, ^62^, ^63^, ^64^ ^]^ However, there are also studies highlighting the negative environmental impacts of technology, such as emissions and e‐waste, emphasizing the potential conflicts between DT and GT.^[^ ^65^, ^66^, ^67^, ^68^, ^69^ ^]^

Institutional quality plays a significant role in the link between governance, green growth, and sustainable technological innovation.^[^ ^70^, ^71^ ^]^ The capacity to balance economic growth and environmental well‐being varies across regions, with high levels of prosperity sometimes leading to negative environmental impacts. Emerging economies often focus on growth over environmental conservation.^[^ ^72^ ^]^ While rapid urbanization can bring economic benefits, it may also intensify ecological challenges, requiring mechanisms for sustainable development.^[^ ^73^, ^74^, ^75^ ^]^ The quality of regulations, corruption control, and the rule of law are crucial factors in advancing sustainability.^[^ ^70^ ^]^ The path to sustainability in peripheral regions depends on a mixture of factors, including asset fragility, connectedness, network positionality, socio‐spatial unevenness, and multi‐scalar embeddedness of transition policies.^[^ ^76^, ^77^ ^]^

The penetration of digital means into various sectors necessitates institutional reform and digital leadership. Sustainability depends on reliable institutions, especially in data‐based governance.^[^ ^78^ ^]^ Governance theories stress the relationships between hierarchies, markets, networks, and often focus on political factors, path dependence, and leadership styles. Governments have a crucial task in creating policy coherence, action legitimacy, and consensus among subordinate structures.^[^ ^79^ ^]^ Public management is a key driver of urban transformation toward sustainability, while smart integrative governance is indispensable for achieving the targets set in the SDGs.^[^ ^80^, ^81^ ^]^

The division between core and peripheral regions within the EU also aligns with the disparities in energy transitions, particularly in terms of energy poverty, and calls for differentiated actions in response to these varying needs.^[^ ^80^ ^]^ These inequalities are further exacerbated by political tensions, corruption, and clientelism, which can delay recovery programs and hinder economic growth and green development, particularly in Eastern EU countries.^[^ ^54^, ^82^, ^83^, ^84^, ^85^ ^]^ Emerging economies can focus more on achieving economic growth while the availability of resources can enable regions to accelerate eco‐digitalization to combat pollution and mitigate the impact of climate change.^[^ ^77^ ^]^ As revealed by Bianchini et al.,^[^ ^54^ ^]^ environmental technologies can have a positive impact in regions with advanced digital capabilities, but strategic adaptations are needed based on the innovation and technological capacity of the region, and attention must be given to the environmental costs of digital transformation.

While cities and regions may differ in their approaches, institutions, and governance are instrumental in shaping sustainability. By identifying ways to respond to environmental challenges, both at the urban and regional levels, and complementing these efforts with financial support and policies favoring sustainable development, a positive chain reaction can be initiated. Effective urban planning can attract and propagate good practices to other areas, creating a more sustainable spatial articulation from which communities can benefit.^[^ ^86^, ^87^, ^88^ ^]^

In summary, achieving green and digital transitions requires effective institutions, sound governance, smart planning, and coordinated efforts at various levels. The literature supporting this proposition is vast but rather heterogeneous. Therefore, it is pertinent to focus on the main forces at work, as suggested in Figure 1. This calls for a structured quantitative analysis, which will be pursued in Section 2.

## 2 Methodological Design and Empirical Application

### 2.1 Research Framing

The main purpose of this study is to identify critical factors in the implementation of green and digital transitions within European regions while considering the impact of various socio‐economic and institutional indicators. In alignment with this overarching goal, the research encompasses two key objectives. First, it seeks to present the dynamics of digitalization and ecological transition within the EU, examining the period before and after the COVID‐19 pandemic. Second, it aims to evaluate the progress made in these two domains by analyzing the NRRPs and the financing provided by the RRF. The focus is on countries that have allocated at least 3% of their GDP to these transitions. Specifically, this group includes Greece, Romania, Croatia, Bulgaria, Portugal, Slovakia, Poland, Spain, Latvia, Slovenia, Lithuania, Hungary, Estonia, and Czechia, which are home to lagging regions characterized by low income and low growth, primarily situated in the East and South of the EU. These regions often grapple with adverse sectoral structures, lower skill levels among the resident workforce, limited innovation, and inferior activity rates compared to national and EU averages. These regions will be compared to Northern and Western EU regions.

To assess the “twin” (GT and DT) transition, the scrutiny employs a range of variables and indices that capture the evolution prior and after the onset of the COVID‐19 pandemic. It also incorporates data on the Recovery and Resilience Plans implemented by EU member countries, recognizing their role as potential drivers of these transitions.

This study's more extensive part explores the differentiations at the regional (NUTS2) level regarding green and digital transitions using various available indicators in Appendix 1 and 2 (Supporting Information). It uses XLStat2019 software for applying multivariate analysis, index categorization, correlation analysis, and multiple linear regression (MLR) to test and assess the relation between factors related to the “twin” transition and socio‐economic and institutional ones, that may influence these transitions and explain the regional disparities linked to these interdependent processes. **Table** **1** includes the descriptive statistics of all variables and indices that were integrated in research. The analysis included 236 EU regions divided into selected regions (Eastern and Southern EU) (118 NUTS2 in total – 59 in each geographic area) and non‐selected regions (Western and Northern EU) (118 NUTS2). The pre‐processing of data comprised the choise of indicators and the test of their scale reliability by using Cronbach's Alpha (values between 0.7‐0.9 are reliable) and Spearman's rho (0,3‐0,9).^[^ ^89^ ^]^ Then, the outliers were calculated as values that lay outside 1.5 times the IQR (Interquartile range) from 1^st^ quartile and 3^rd^ quartile. Outliers were trimmed to the nearest value that is not an outlier. Finally, data transformation and normalization were made by rescaling from 0 to 1 using the Min‐Max function.

**Table 1:** Variables used in the empirical analysis – descriptive statistics.

Statistics	1st Quartile	Median	3rd Quartile	Mean	Variance (n‐1)	Std. deviation (n‐1)
GHG emissions	0.105	0.175	0.280	0.217	0.030	0.172
GHG emissions growth rate	0.406	0.465	0.529	0.471	0.014	0.118
Premature deaths attributed to PM_2.5_	0.279	0.365	0.473	0.382	0.031	0.175

RETI	**0.460**	**0.554**	**0.714**	**0.586**	**0.032**	**0.178**
Individuals who use the Internet for interaction with public authorities	0.497	0.655	0.857	0.645	0.055	0.236
Households with broadband access (%)	0.879	0.909	0.929	0.899	0.006	0.076
Individuals buying over the Internet the last year	0.589	0.742	0.828	0.707	0.031	0.176
Access to high‐speed broadband	0.033	0.320	0.599	0.363	0.104	0.323
Individuals with above‐basic overall digital skills	0.318	0.523	0.692	0.524	0.059	0.243
Enterprises having received orders online	0.200	0.218	0.447	0.291	0.037	0.192
Enterprises with fixed broadband access	0.553	0.737	0.868	0.623	0.083	0.289

RDTI	**0.314**	**0.398**	**0.560**	**0.436**	**0.030**	**0.173**
Population density	0.068	0.115	0.186	0.150	0.018	0.133
Gross domestic product	0.175	0.259	0.365	0.283	0.024	0.155
Healthy life expectancy	0.459	0.684	0.823	0.621	0.065	0.255
Higher educational attainment	0.414	0.520	0.648	0.534	0.027	0.165
Employment rate	0.575	0.707	0.833	0.680	0.042	0.204
Innovative SMEs	0.166	0.336	0.400	0.302	0.024	0.156
Total patent applications	0.008	0.035	0.115	0.096	0.024	0.155
Core creative class employment	0.353	0.441	0.559	0.471	0.029	0.171
Control of corruption	0.368	0.559	0.768	0.558	0.058	0.240
Quality and accountability	0.389	0.591	0.709	0.542	0.046	0.214
Corruption in the local or regional public institutions	0.443	0.607	0.820	0.619	0.057	0.238

First, our approach proposes two aggregate indices, one related to the environmental transition and the other a proxy for digital transition. Each index included various indicators, and the methodology for weighing these variables implied the application of PCA/FA and the use of factor loadings and explained variance.^[^ ^89^, ^90^, ^91^ ^]^ Each stage of the process is detailed in Appendix 3 (Supporting Information).

The two indices are as follows:

- The Regional Environmental Transition Index (RETI) primarily focuses on the carbon transition and reducing the effects of air pollution on the population's health using as proxies GHG emissions and premature deaths attributed to fine particulate matter pollution (PM_2.5_) (**Table** **2**).
- The Regional Digital Transformation Index (RDTI) encircles seven indicators related to individuals' and enterprises' use of technology and the Internet, including Internet transactions and interactions with public authorities.

**Table 2:** The indicators included in RETI and RDTI and their weights.

	Wk [weight]
RETI	
Global warming potential (GHG emissions)	0.410
Global warming potential (GHG emissions growth rate)	0.350
Premature deaths attributed to PM_2.5_	0.240

RDTI	
Access to high‐speed broadband	0.350
Households with broadband access [%]	0.160
Individuals who use the Internet for interaction with public authorities	0.130
Individuals buying over the Internet the last year	0.110
Individuals with above‐basic overall digital skills	0.100
Enterprises having received orders online	0.080
Enterprises with fixed broadband access.	0.070

Second, a correlation analysis was performed to highlight the significance of the relations between RETI and RDTI on one side, and various socio‐economic and institutional indicators, on the other. These were tested in the case of the 118 selected regions from Southern and Eastern EU and in all 236 regions of the EU included in the database, to emphasize the specific patterns of our interest area. Detailed information on the correlations is presented in Appendix 4 (Supporting Information).

Third, the regression analysis was performed for all EU regions and by geographic area to model the differentiated processes and drivers that influence green and digital transition (RETI and RDTI), which were the dependent variables that were tested against all socio‐economic and institutional variables described in Appendix 2 (Supporting Information). The approach uses the best model that retained a number of maximum five explanatory variables given the R^2^ (min. 0.544; max. 0.690), the p‐value of the F statistic computed in the ANOVA table, and the significance level of 5%. Based on the Type III sum of squares, the variables that bring the most momentous information were selected to explain the changeability of the dependent variable. Statistical tests were accomplished to validate the models (see Appendix 5, Supporting Information).

Finally, complementary visualization techniques were also employed in Arc GIS Pro (for RETI and RDTI) to underline the spatial differentiations and illustrate the territorial patterns of both, using a bivariate color matrix. The three‐variated connection between RETI, RDTI, and distinctive institutional indicators was graphically illustrated using Zenplot software, showing the discrepancies between selected regions from Eastern and Southern Europe and the rest of the EU regions.

Our study seeks to tackle the following key directions: the dynamics of green transition and digitalization (sub‐Section 2.2), the (dis)similarities (sub‐Section 2.3), and the determinants of the green and digital transition in EU regions (sub‐Section 2.4) seen from the perspective of institutional quality.

### 2.2 The Dynamics of Green Transition and Digitalization within the EU

This section explores the diverse paths taken by EU countries and regions in their journeys toward GT and DT before and during the COVID‐19 pandemic. The GT is intricately linked to enhancing environmental performance, driven by innovations and structural shifts in economic activities. Such changes can foster not only increased opportunities through cleaner energy, sustainable materials, and eco‐friendly production techniques but also the reduction of waste. This transformation necessitates a systemic shift, decoupling GDP growth from material consumption, minimizing the ecological footprint of materials, and influencing consumer choices to create a new growth model that underpins economic competitiveness and well‐being and ensures a safe operational space within the Earth's ecological limits.^[^ ^92^ ^]^ The more the sources of renewable energy are diversified, the more, in principle, the ecological footprint deepens.^[^ ^93^ ^]^ If, in addition, the institutions are performing well and the promoted policies are aimed at a sustainable environment, the positive effects will not be long coming.^[^ ^94^, ^95^ ^]^

The GT can be viewed from various angles, with a multitude of indicators and composite indices reflecting different components of this transition. For instance, the Environmental Performance Index (EPI), introduced by Yale University in the 1990s, amalgamates various indicators within several dimensions at the national level. The environmental health dimension (comprising air quality, health sanitation and drinking water, heavy metals, and waste management) reflects relatively modest transformations, with incremental improvements in all regions. In contrast, ecosystem vitality displays more noticeable differences, with greater improvements in Southern Europe, particularly in marine area management, while Western Europe records more modest progress. Overall, over the last decade, EU countries have shown positive evolution across all dimensions, with more pronounced advancements in Northern Europe compared to the East and West.^[^ ^96^ ^]^ A critical facet of the GT focuses on combating climate change. Despite some improvements, noteworthy variations exist between Northern Europe and other European regions, predominantly in terms of emissions reduction and the adoption of renewable energy sources. Another index, the Environmental Transition Performance Index (ETPI), considers four separate components. Among these, energy and material productivity have displayed the most prominent positive trends, while emissions reduction and biodiversity recorded more limited advancements from 2011 to 2020. This data paints a slightly different picture on a European scale, with Nordic countries ranking lower in the hierarchy and Mediterranean countries (such as Italy and Malta) performing notably above the EU average. Generally, countries showing improvements in energy productivity and material usage have made the most rapid strides in the GT.

To illustrate the DT, a composite index was used, namely the Digital Economy and Society Index (DESI), that summarizes relevant indicators about digitalization, distinguishing between regions in the eastern part of Greece (severely affected by the 2008 economic crisis) and former communist countries, which, with the exception of the Baltic countries, exhibit a relative lag and lower performance, particularly in terms of human capital and the integration of digital technology. The superior performance of Nordic countries, the Netherlands, Spain, and Ireland, stand out. Further analysis of individual indicators found in the Digital Development Dashboard (DDD) corroborates this, with Nordic countries leading in fixed broadband subscriptions, active mobile broadband subscriptions, and internet usage, and Eastern and Southern Europe exhibiting the lowest performances. Notably, the pandemic brought about some changes in this hierarchy, even though differences in access to the internet, especially urban‐rural disparities, still remain a matter of concern.

### 2.3 NRRPs – (Dis)Similarities in Planning Green and Digital Transition

**Figure 2:**
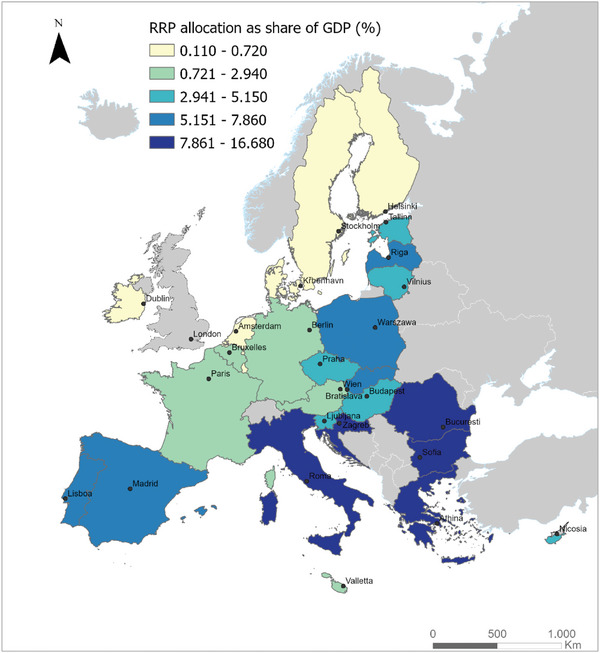
RRF allocations (% of GDP) in EU countries Source: authors’ representation based on Bruegel assessment data^[^ ^111^ ^]^ and European Commission.^[^ ^112^ ^]^

The preceding section outlined the disparities in GT and DT at the national level. In the current post‐pandemic context, the NRRPs play a pivotal role in accelerating these transitions within EU countries, ensuring the efficient and effective use of allocated funds. The choices made by EU member states have shown a high degree of variance (see **Figure** **2**). While some countries have allocated substantial funds to both the GT and DT (e.g., Belgium, Ireland, Austria), others have predominantly centered on one of these transitions (e.g., Denmark, Romania, Poland for GT; Latvia, Greece for DT). The choice of principal focus often corresponds to the level of support, with countries receiving lower RRF funds as a share of their GDP in the digital sector. Generally, countries tend to allocate more to reducing greenhouse gas emissions.

**Figure 3:**
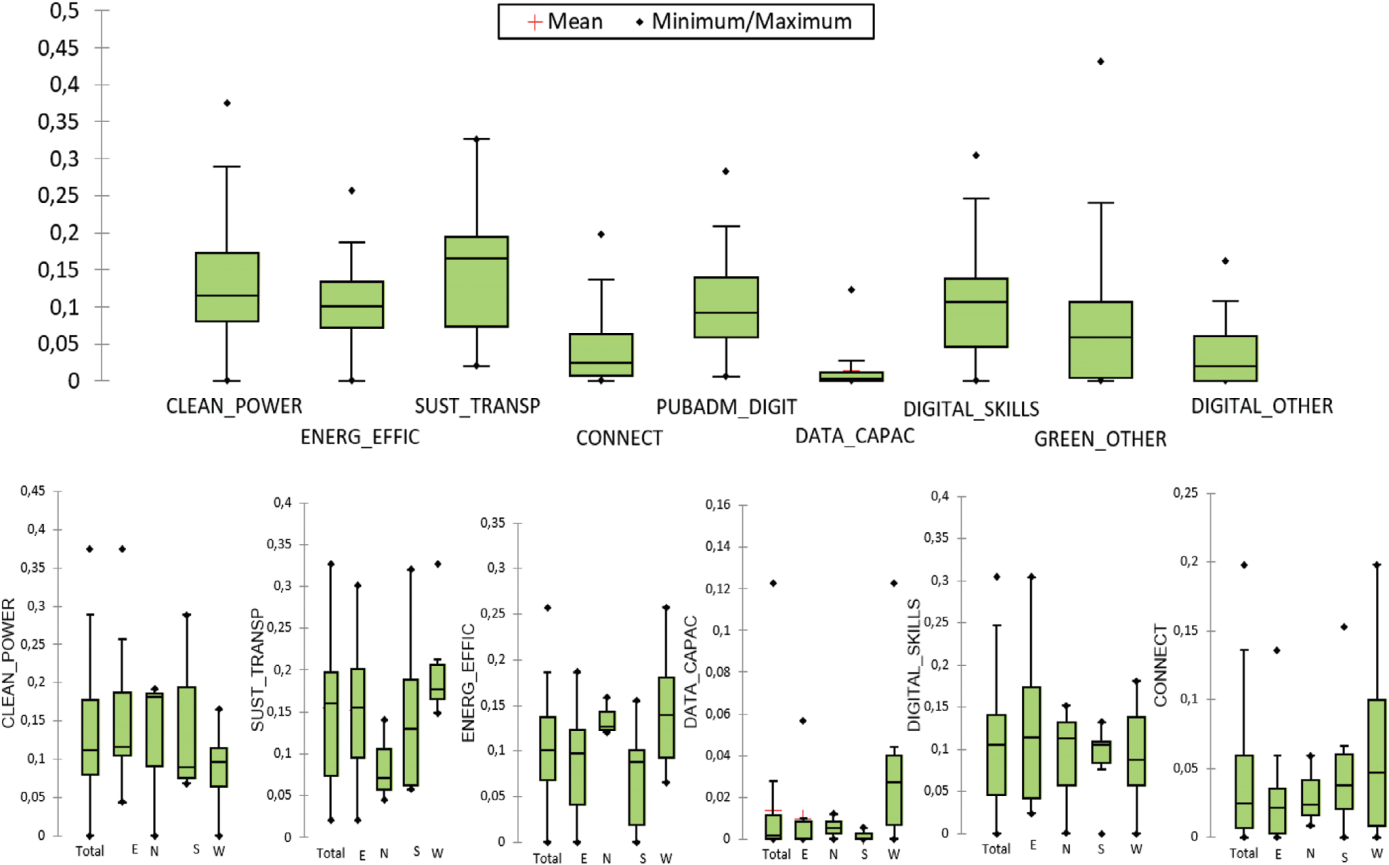
Flagship areas for investments and reforms *Source*: authors’ representation based on and Bruegel assessment^[^ ^111^ ^]^ and European Commission.^[^ ^112^ ^]^ *Note*: E = East; N = North; S = South; W = West.

Moreover, the specific flagship areas for investments and reforms provide insights into the direction of investments, with a concentration on clean energy (CLEAN_POWER) and sustainable transport (SUST_TRANSP) on the one hand, and the digitalization of public administration (PUBADM_DIGIT) and digital skills (DIGITAL_SKILLS) on the other (see **Figure** **3**).

A closer examination emphasizes geographical disparities. In the green flagship areas, there is a relatively homogeneous distribution across the EU, with somewhat reduced allocations in Western countries, which have previously invested more in energy efficiency (ENERG_EFFIC), particularly the Nordic countries. In most of the analyzed states, SUST_TRANSP is also prioritized, although Nordic Europe is already financing and implementing initiatives in this regard from alternative sources. The digital indicators paint a similar picture for PUBADM_DIGIT, where Nordic Europe has taken significant strides using other funding sources. Northern and Southern Europe are inclined toward rapid broadband service deployment (CONNECT), while Eastern Europe continues to face challenges in this field.

A correlation analysis of these variables (see **Table** **3**) reveals how investments in CLEAN_POWER associate negatively with those in ENERG_EFFIC, indicating that countries prioritizing clean energy may not have allocated equivalent resources for enhancing energy efficiency. On the other hand, allocations for ENERG_EFFIC are positively connected with investments in data cloud capacities and sustainable processors (DATA_CAPAC) and education and training to support digital skills (DIGITAL_SKILLS).

**Table 3:** Correlation between the financial support for different flagship areas.

Variables	CLEAN_POWER	ENERG_EFFIC	SUST_ TRANSP	CONNECT	PUBADM_DIGIT	DATA_CAPAC
CLEAN_POWER						
ENERG_EFFIC	**−0.446**					
SUST_TRANSP	−0.065	**−0.170**				
CONNECT	0.045	−0.058	**0.194**			
PUBADM_DIGIT	0.107	−0.090	0.028	−0.103		
DATA_CAPAC	**−0.154**	**0.597**	**0.327**	**0.279**	**−0.190**	
DIGITAL_SKILLS	−0.010	**0.289**	**−0.327**	−0.110	**−0.297**	**0.165**

*Values in bold are different from 0 with a significance level alpha = 0.05*

This brief assessment is mainly at the national level and doesn't exclude persistent inter‐regional disparities in the “twin” transition. Nevertheless, in the less advanced regions, efforts are condensed on increasing investment to bridge gaps in digital skills.

### 2.4 Drivers of Green and Digital Transition in the EU

**Figure 4:**
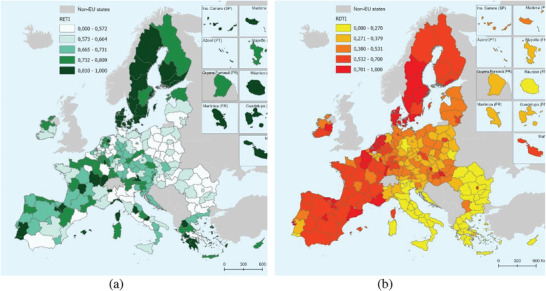
Regional Environmental Transition Index (RETI) a) and Regional Digital Transformation Index (RDTI) b) (authors’ representation).

In our pursuit to distinguish progress in GT and DT at the regional level, the two aggregate indices proposed (RETI and RDTI) reveal a somewhat contrasting image. Regarding RETI, an intricate mosaic unfolds, reflecting substantial inter‐regional gaps (see **Figure** **4**a). These are not solely attributed to varying policy measures but are equally influenced by the regions' diverse natural and economic potential. Less densely populated and relatively isolated regions may make transitioning toward a reduced climate footprint easier. On the other hand, regions with dense populations and extensive industrial activities, such as the metropolitan areas of major European cities, exhibit reduced environmental performance, underscoring the fact that the separation between economic growth and environmental quality remains problematic.

A substantial cluster of regions in Northern Europe exhibits superior performance, particularly in efficiently reducing atmospheric emissions, which is partly associated with their smaller populations. Conversely, RDTI presents a more uniform territorial distribution, offering a clearer differentiation. Regions in the Southeast of the EU, namely Romania, Bulgaria, Greece, and Southern Italy (Mezzogiorno), generally display lower performance, with capital cities being notable exceptions (see Figure 4b).

**Figure 5:**
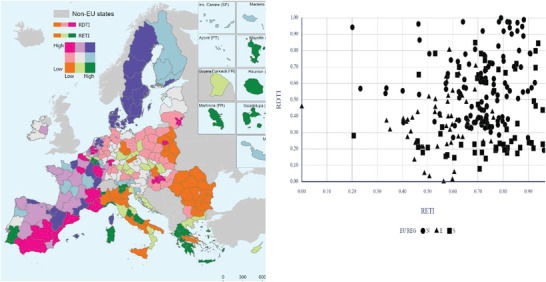
Spatial patterns of green and digital transition in the EU regions (RETI and RDTI, N – Northern EU, E – Eastern EU, S – Southern EU) (authors’ representation).

In contrast, the Northern regions (including Denmark, Sweden, and Finland) and the Western and South‐Western regions (comprising Ireland, France, and Spain) have experienced robust digitalization processes. Therefore, when assessing the twin transition as a whole, beyond the well‐documented dissimilarity between Northern Europe (excelling in both components) and Eastern Europe (excluding the Baltic countries) and, to some extent, Southern Europe (including Greece, Croatia, and Southern Italy), the remainder of the EU displays a relatively heterogeneous regional landscape. These differences result from an accumulation of interdependencies emanating from the regions' inherent potential and the policies implemented at the national and European levels. A comprehensive array of indicators was examined to discover the factors explaining the territorial disparities (see **Figure** **5**).

Some indicators were omitted due to their limited relevance, while others were excluded due to multicollinearity. Ultimately, selected and standardized factors displayed varying correlations with RETI and RDTI.

As a whole, RETI revealed positive links with life expectancy (LIFE_EXP), the quality and accountability of institutions (QUAL_ACCOUNT), and the existence of innovative SMEs (INNOV_SME). However, the studied area exhibited certain peculiarities, including a negative relationship with green jobs, indicating lower ecological performance in these regions. Interestingly, there was a curious positive correlation with local‐level corruption (CORR_LOC_REG), especially with regard to Southern EU. Although this phenomenon aligns with findings in developing countries, where corruption can offer short‐term “flexibility” during transitions, it's essential to recognize that, over the long term, corruption negatively impacts green transition. Most studies emphasize the detrimental effects of corruption on green transition, as highlighted, for instance, by Chatzistamoulou.^[^ ^97^ ^]^

The socio‐economic and institutional indicators presented in Appendix 2 (Supporting Information) appear to exhibit much more powerful links with RDTI. While the influence of population density was relatively weaker, other factors under scrutiny displayed robust connections. The strongest statistical relationships are identified between RDTI and higher educational attainment (HIGH_EDU), along with core creative class employment (CREATIVE), and institutional factor (QUAL_ACCOUNT). GDP exhibited a strong association with RDTI. Notably, regions in Eastern and Southern Europe, characterized by generally lower RDTI values, showed a somewhat less coherent liaison between digitalization and patents (PATENTS), signifying a less advanced phase of digitalization yet to generate major economic and social impacts. The link between life expectancy (LIFE_EXP) and digitalization was less apparent in Southern and Eastern Europe. Instead, superior environmental quality and a reduced ecological footprint appeared to align with higher life expectancy (Western and Northern EU).

**Table** **4** offers an extensive overview of the correlations throughout the EU territory, with geographical distinctions. At the EU level, a positive correlation (0.154) is observed between the two transition indices (RETI and RDTI).

**Table 4:** Correlation matrix values – RETI, RDTI, and selected independent variables.

Variables	All EU		Southern and Eastern EU		Southern EU		Eastern EU		Western and Northern EU	
	RETI	RDTI	RETI	RDTI	RETI	RDTI	RETI	RDTI	RETI	RDTI
RETI		**0.154**		0.124		0.110		0.100		0.013
RDTI	**0.154**		0.124		0.110		0.100		0.013	
POP	**−0.580**	0.115	**−0.562**	0.102	**−0.824**	0.121	**−0.661**	0.011	**−0.621**	**0.185**
GDP	0.063	**0.467**	0.076	**0.365**	**−0.284**	0.160	**0.274**	**0.608**	−0.166	**0.308**
LIFE_EXP	**0.370**	**0.299**	**0.387**	0.025	**−0.336**	−0.051	**0.373**	−0.041	**0.188**	**0.543**
HIGH_EDU	**0.139**	**0.672**	0.082	**0.666**	0.051	**0.801**	0.193	**0.528**	0.066	**0.581**
EMPLOY	−0.047	**0.488**	−0.177	**0.426**	−0.086	**0.417**	**0.321**	**0.673**	−0.175	**0.281**
INNOV_SME	**0.130**	**0.520**	0.134	**0.375**	**−0.349**	**0.267**	**0.307**	**0.585**	**−0.248**	**0.313**
PATENTS	−0.026	**0.311**	−0.040	0.005	**−0.480**	−0.115	**0.403**	**0.366**	**−0.232**	**0.187**
CREATIVE	0.036	**0.587**	−0.121	**0.481**	−0.223	**0.403**	0.209	**0.678**	−0.035	**0.485**
QUALIT_ACCOUNT	**0.318**	**0.542**	**0.414**	**0.380**	0.203	**0.570**	**0.285**	0.118	−0.004	**0.392**
CORR_CONTRL	−0.019	**−0.414**	0.162	**0.442**	0.104	**0.681**	0.157	0.058	−0.002	**0.195**
CORR_LOC_REG	**0.228**	**0.513**	**0.594**	0.167	**0.373**	**0.608**	**0.420**	−0.074	−0.099	**−0.417**

*Values in bold are different from 0 with a significance level alpha = 0.05*.

Next, we adopted a multi‐variate regression analysis (see **Table** **5**), applied to each transition across the entire EU sample (All EU) and the study area (Selected). The models revealed both similarities and particularities. Variables such as population (POP) with higher education (HIGH_EDU), SMEs with innovation cooperation activities (INNOV_SME), and quality and accountability (QUALIT_ACCOUNT) consistently exhibited the most explanatory power for the digital and green transition in both samples. The models retained a maximum of five indicators, using the best model variable selection method. It is evident that both RETI and RDTI are strongly influenced by the considered independent variables, as indicated by R^2^ values, registering 0.575 in the case of RETI and 0.555 for RDTI for the EU level models and 0.703 for RETI and 0.611 for RDTI, in the case of the selected regions (Southern and Eastern Europe). At a significance level of 5%, the information provided by the explanatory variables notably outperforms what the basic mean would offer in all four cases.

**Table 5:** Empirical regression models.

Indices	Study area	Regression model	MSE	R^2^	Adj. R^2^	Mallow' Cp	Akaike's AIC	Schwarz's SBC	Amemiya's PC
**RETI**	Selected	0.334‐0.701*POP‐0.152*EMPLOY +0.339*INNOV_SME+0.194*QUALIT_ACCOUNT+0.464*CORR_LOC_REG	0.008	0.703	0.690	7.728	−559.092	−542.468	0.323
	All EU	0.449‐0759*POP +0.233*LIFE_EXP +0.092*HIGH_EDU+0.119*QUALIT_ACCOUNT+0.145*CORR_LOC_REG	0.011	0.575	0.566	5.612	−1059.766	−1039.008	0.443
**RDTI**	Selected	−0.294‐0991*GDP‐0.379*LIFE_EXP +0.796*HIGH_EDU+1.788*INNOV_SME+0.449*CORR_LOC_REG	0.017	0.611	0.594	21.967	−472.112	−455.487	0.423
	All EU	−0.157‐0.549*GDP +0.757*HIGH_EDU +0.200*EMPLOY+0.627*INNOV_SME+ 0.117*QUALIT_ACCOUNT	0.024	0.555	0.545	4.544	−869.314	−848.557	0.464

In general, RETI is shaped by the presence of corruption in a country's local or regional public institutions (CORR_LOC_REG) and institutional quality (QUALIT_ACCOUNT). This could also involve a more significant negative environmental impact. Nevertheless, the role of institutions remains highly relevant. For RDTI, SMEs innovations (INNOV_SME) and higher education (HIGH_EDU) emerged as more prominent drivers of digitalization (Table 5).

**Figure** **6** illustrates the relationship between the RETI, RDTI, and the institutional factors (control of corruption, impartiality, and quality and accountability). The distinction between the selected regions and the rest of the EU regions is relatively clear. However, there exists a “mixed area” of Southern or Eastern regions that perform exceptionally well in both transitions and institutional performance, alongside less‐performing regions in Northern or Eastern Europe.

Figure 6The triple relation between institutional indicators (a – corruption, b – impartiality, c ‐quality and accountability), RETI, and RDTI (authors’ representation) *Note*: the larger the circles, the higher the institutional quality; Y = selected regions (Eastern and Southern EU regions), N = non‐selected regions (Western and Northern EU regions).

**Figure:**
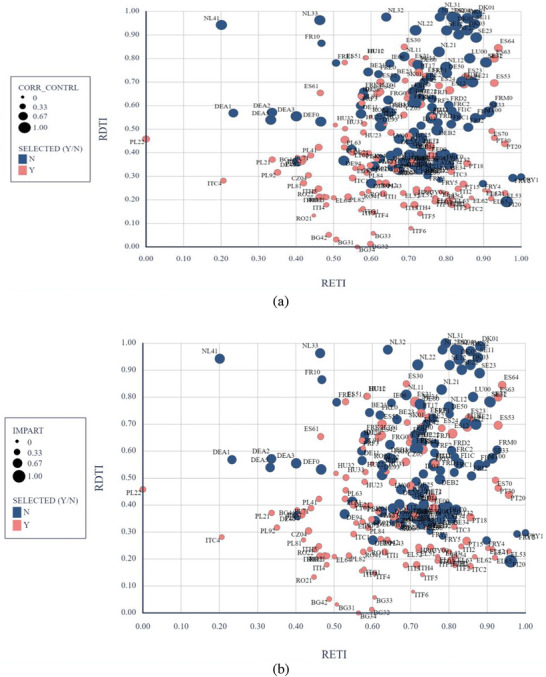

**Figure:**
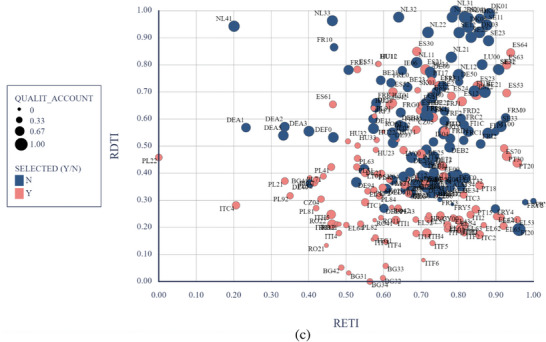

The regions are labeled as having varying levels in terms of the implementation of digital technologies, and, largely, those left behind face challenges in quite a batch of directions, including economic or environmental ones. It is unfeasible to achieve an alignment of regional smart development, given that the factors that propel it have different intensities within each region.^[^ ^98^ ^]^ Therefore, it seems that the main tendency is to register scale effects on digitalization in competitive regions and in urban agglomerations, where innovation is concentrated. In contrast, less developed regions experience the effect of domino, markedly if their institutional system is malfunctioning.

In summary, despite the noticeable Northwest‐Southeast gradient observed for the three components – green, digital, and institutional – and the distinctive territorial patterns at a macro‐scale, the interregional differences within countries are increasingly significant and warrant targeted policy attention.

### 2.5 Institutional Performance and Successful “Twin” Transition

This study strongly supports the hypothesis that the convergence of green and digital transitions is closely tied to institutions' quality and ability to develop and implement sustainable strategies in both the medium and long term. Based on the results of our analysis, it becomes evident that responsive and accountable governance should serve as the foundation when discussing both transitions. The parallel advancement of digitalization and sustainability is imperative in our contemporary times. While numerous models of government intervention can be proposed in theory, practical experience shows that digital transformation necessitates, first and foremost, digital skills among employees and leaders. It also requires concentrated efforts in regions with low innovation capacity, corruption, lack of political commitment, inadequate leadership, poor service quality, and ineffective means of identifying issues within the territory.^[^ ^57^, ^99^ ^]^ Once these elements are addressed, digital technology projects can continuously emerge, becoming the connecting thread toward a more eco‐friendly society and guiding decision‐making toward the common good through open and transparent policies, institutional robustness, and a culture of learning, for developing predictable evolution scenarios.^[^ ^100^, ^101^ ^]^

The acceleration of digital and green transitions can be associated with a model based on the Common Assessment Framework (CAF), which can play a significant role in enhancing institutional capacity when key factors (i.e., the quality of leadership, innovative management, partnerships in advancing digitalization, engaged and productive individuals) are adequately leveraged.^[^ ^102^ ^]^ Essentially, digital governance can be a tool that supports sustainable development.^[^ ^103^ ^]^ While digitalization can positively shape the GT, it is noted that, compared to the socio‐economic aspect, the influence of digitalization on GT is relatively smaller. To increase its impact, sub‐national leaders should ensure that there are resources for absorbing, utilizing, and proactively addressing future challenges.

Countries should act according to internationally established standards, such as the UN SDGs, to comprehensively assess sustainability on multiple levels. The SDGs emphasize the relationship between smart technologies and environmental performance, underscoring that success in achieving sustainable economic and environmental objectives hinges on the quality of government and regulations, readiness to innovate, investment capacity, willingness to adapt, the active participation of individuals in management structures, and the motivation to effect positive change.^[^ ^104^ ^]^

The regional economic dispersion has implications on the absorption potential of technologies that require low carbon emissions. The less developed regions encounter, most of the time, difficulties when it comes to boosting research related to environmental protection and ecology, attracting skilled and creative human resources to put into practice innovative ideas for the green economy, or making public and private investments to allow the alignment of policy objectives with local specifics. All these also spread out in the neighboring regions, highlighting a trend of low capacity for managing environmental issues, which is reflected, in the long term, in the quality of people's lives (i.e., deaths associated with PM_2.5_ or decrease in life expectancy). Then, the plentiful negative consequences that global warming brings, both on humans and on plant and animal species, deepen the vulnerabilities.

Institutionally, environmental regulations should be known by the large public and in order for the policies in this field to have the expected impact, consistent campaigns have to be advanced by governments, educational institutions, NGOs, and think‐tanks to promote the importance of environmental protection, renewable energy, or biodiversity conservation. Green education must be furnished from the first years of school to have responsible citizens. Civic action can be enhanced through regular public consultations, by running, for example, competitions between regional teams for finding innovative solutions for an eco‐friendly environment. Representatives from the public administrations can also participate in these, to discuss the proposed solutions and transpose them into normative documents. Afterwards, the existence of dashboards with regional data or comparative maps that define sustainability can be brought to the attention of citizens so as to draw remedial measures for those indicators that constitute the so‐called alert systems, which require urgent intervention.

Our findings suggest that, at the level of EU regions, there is greater heterogeneity in terms of environment compared to digitalization. The European regional statistics on the digital dimension are not plentiful, most referring to the Internet connection, the purpose for which this is used, and the digital skills. In the case of environmental issues, they include various angles, from global warming, biodiversity, waste management, water, and air quality, to natural hazards, existing several measurements and indicators that define them. In subsequent work, we intend to expand the space of analysis, addressing the green and digital transition beyond the European continent, precisely to ascertain whether Europe aligns itself with the UN SDGs targets and where it compares with international competitors. In addition, given that with the implementation of the EGD objectives, multiple challenges arise in relation to governance, it would be interesting to explore how the states move toward climate neutrality, the trade‐off between economic growth and environment, fair digital and green transition, resilience and sustainability, all accompanied by analyzes regarding the financial implications. All in all, a better future can be ensured by fully considering environmental and digital issues.

## 3 Conclusion

The conclusions drawn from this study emphasize the enduring heterogeneity of the European landscape, where the digital divide and sustainability significantly impact resilience capacity. There is a clear situation of left‐behind ones.^[^ ^105^ ^]^ Bridging these gaps, especially when economic, social, and environmental disparities are interconnected with an inflexible institutional framework, is a formidable challenge. To address this issue effectively, a people‐centric approach to technological innovation that integrates environmental responsibility and digital literacy is essential. The implementation of the RRF through NRRPs serves as an opportunity for convergence, fostering short‐term competitiveness through digitalization and long‐term sustainability through the GT, particularly for countries encompassing low‐income regions (Eastern EU) or regions with low economic growth (Southern EU). In contrast, the stakes for wealthier nations in the Northern and Western EU are relatively lower.

Southern and Eastern Europe, which receive a significant portion of NRRPs funds, exhibit noticeable disparities compared to the rest of the EU in numerous aspects, and urban‐rural disparities are even more pronounced. These discrepancies can grow if investments are directed toward already developed regions with high absorption capacity, leaving less‐prepared regions further behind. Thus, the interconnected nature of GT and DT implies that a systemic approach is necessary, beyond specific investments in environmental protection or ICT. The success of recovery and resilience funds depends on accounting for varying territorial capacities at the national and regional levels. The institutional component plays a pivotal role in perpetuating the differences between Northern and Western Europe, and Southern and Eastern Europe. Positive progress in both digitalization and the GT is intrinsically linked to the control of corruption, impartial institutions, and accountable governance. Particularly, digitalization appears to be more heavily influenced by institutions, but a clear connection can also be observed in the case of the GT.

Many projects and reforms within NRRPs are not integrated into a comprehensive vision but rather take advantage of available funds, sometimes leading to an emphasis on traditional transport infrastructure, such as highways and railways, under the green transition banner. A more determined approach is needed, especially given that the recent COVID‐19 pandemic and the war in Ukraine have pushed back progress toward SDGs in several areas.

The minimum thresholds for green and digital measures in NRRPs have created proportionality between the two, revealing that many countries prioritize other areas, including social, economic, and institutional development. Countries with a higher share of their GDP allocated to RRP funds can accelerate both transitions, potentially exacerbating regional disparities. The allocation of RRP funds can create competition rather than convergence between green and digital actions, indicating a lack of long‐term perspective, particularly in the East. Therefore, additional analyses on regional sensitivity should be conducted once more data becomes available, as many projects and reforms are challenging to locate and analyze for their differential impact on regions requiring integrated support. After conducting our analysis, three predominant approaches can be identified:

- The *path dependency approach* – RETI and RDTI reveal an East‐West divide, with the East experiencing significant inter‐regional disparities;
- The “*filling the gap*” *approach* – prioritizing regions with high deficiencies or negative dynamics;
- *Heterogeneous approaches* – investments diverge across multiple areas, with some regions adopting focused or opportunistic approaches, prioritizing domains perceived as flagships to enhance competitiveness.

The choices made by EU member countries and the allocations at the regional level reflect a desire either to reduce gaps between the two domains or to strengthen pre‐existing capacities. Territorial units that excel in addressing the intersection of digital and green issues tend to possess above‐average human capital and training capacity, particularly in research and development (R&D).

The decision‐makers should work in an integrated way to propagate the beneficial effect of the policies in more extensive areas. When the reference is made to pollution or climate warming, they go beyond borders and, therefore, solidarity matters in the success of policies. Transitions of whatever nature need social validation and involvement, and governments have to be concerned with sustainable and just policies.^[^ ^106^ ^]^ The acceleration of the digital and green transition can be induced through coherent policies, the results of which will be better observed with the progress in achieving the sustainable development objectives included in the EGD.^[^ ^107^ ^]^ Although NGEU stands for sustainability and the RRF represents a relevant tool for constructive transformations, it remains to be seen whether digital and green transitions, as mentioned in EU strategies, will gain depth by the end of the current programming period or will remain only simple constructs or too high aspirations. Equally, Horizon Europe calls can contribute to the vitalization of peripheral regions, especially in the eastern part of the EU, and the dissemination of information about these opportunities is critical for the initiation of projects that will give an impetus to green and digital investments.^[^ ^108^ ^]^ Otherwise, the less developed regions risk distancing themselves even more from the advanced ones, giving way to a more polycentric Europe.^[^ ^109^ ^]^

In conclusion, transitioning to a digital economy is associated with higher‐performing institutions and a lower level of corruption (see also^[^ ^110^ ^]^). Moreover, education and the acquisition of skills in new technologies will provide essential support for both green and digital transitions. Clearly, spatial imbalances call for effective institutional support mechanisms at the regional scale.

## Conflict of Interest

The authors declare no conflict of interest.

## Supporting information

Supporting Information

## Data Availability

The data that support the findings of this study are available in the supplementary material of this article.
