# Supplementary material for: Institutional Quality in Green and Digital Transition of EU Regions – A Recovery and Resilience Analysis
Source: Glob Chall. 2024 Aug 20;8(9):2400031. doi: 10.1002/gch2.202400031 (PMC11492339; doi:10.1002/gch2.202400031)
Supplement: Supplementary file 1 — Supporting Information [file GCH2-8-2400031-s001.docx]

**INSTITUTIONAL QUALITY IN GREEN AND DIGITAL TRANSITION OF EU REGIONS –**

**A RECOVERY AND RESILIENCE ANALYSIS**

**Alexandru Bănică**, Center for Geographic Research, Romanian Academy, Iasi - Romania

**Ramona Ţigănaşu,** Centre for European Studies, “Alexandru Ioan Cuza” University of Iasi, Romania

**Peter Nijkamp,** Open University, Heerlen, the Netherlands

**Karima Kourtit,** Open University, Heerlen, the Netherlands

APPENDIX 1. INDICATORS USED IN COMPOSING RETI & RDTI

| **Indicator name** | **Description** | **Measurement** | **Source** | **Reference year** | **Geographical level** |
| --- | --- | --- | --- | --- | --- |
| Global warming potential (GHG emissions) | Emissions of total greenhouse gases (GHG) include CO2 (from fossil sources only), CH4, N2O, and F-gases. They are expressed in kilotons of CO2 equivalent (CO2eq). CO2eq emissions are calculated using the Global Warming Potential values from the Fourth Assessment Report of the Intergovernmental Panel on Climate Change (GWP-100 AR4). | Kton CO2 equivalent/GDP | EDGAR (Emissions Database for Global Atmospheric Research) | 2021 | NUTS 2 |
| Global warming potential (GHG emissions growth rate) | Total greenhouse gas (GHG) emissions include CO2 (from fossil sources only), CH4, N2O, and F-gases. CO2eq emissions are calculated using the Global Warming Potential values from the Intergovernmental Panel on Climate Change's Fourth Assessment Report (GWP-100 AR4). | % | EDGAR (Emissions Database for Global Atmospheric Research) | 2012-2021 | NUTS 2 |
| Premature deaths attributed to PM2.5 | Premature deaths attributed to PM2.5 in 2019 at the NUTS3 level in European countries. | no. of deaths per 100,000 inhabitants | European Environmental Agency | 2020 | NUTS 3 aggregated in NUTS 2 |
| Households with broadband access | Broadband access in households | % of total households | Eurostat regional information statistics (code: isoc_r_broad_h) | 2021 | NUTS 2 |
| Individuals buying over internet the last year | Percentage of individuals who ordered goods or services over the internet for personal use in the last 12 months. | % of individuals | Eurostat Regional Information Statistics (code: isoc_r_blt12_i) | 2021 or last available year | NUTS 2 |
| Individuals who used the internet for interaction with public authorities | Percentage of individuals who reported using the internet to interact with public authorities in the last 12 months | % of respondents who agree | Eurostat (code: ISOC_R_GOV_I) | 2021 | NUTS 2 |
| Individuals with above-basic overall digital skills | Individuals with above-basic overall digital skills converted to an index relative to the EU average in 2021 (EU average = 100) | Index of EU-27 2021  (EU-27 average = 100) | Regional Innovation Scoreboard, DG Internal Market, Industry, Entrepreneurship and SMEs and DG Regional and Urban Policy elaboration | 2020 | NUTS 2 |
| Enterprises having received orders online (at least 1 %) | % of enterprises with at least 10 persons employed in the given NACE sectors, by size class (NACE Rev 2 since 2009), that have received orders online (at least 1%) | % of enterprises | Eurostat Community Survey on ICT usage and e-commerce (code: tin00111) | Average 2019–2021 | NUTS 0, NUTS 2 |
| Enterprises with fixed broadband access | Enterprises connected to an exchange that has been upgraded to support xDSL technology, a cable network upgraded for internet traffic, or other broadband technologies. This includes both fixed and mobile connections. | % of enterprises | Eurostat Community Survey on ICT usage and e-commerce (code: tin00090) | Average 2016–2017 | NUTS0, NUTS 2 |

APPENDIX 2. SOCIO-ECONOMIC AND INSTITUTIONAL INDICATORS

| **Pillar name** | **Indicator name** | **Description** | **Measurement** | **Source** | **Reference year** |
| --- | --- | --- | --- | --- | --- |
| Socio-economic | POP:  Population density | Demographic data at regional level include statistics on the population at the end of the calendar year. | Inhab./km^2^ | Eurostat (code: demo_r_pjangrp3) | 2021 |
|  | GDP:  Gross domestic product | Gross domestic product (GDP) in volume by NUTS 2 regions | Mil. Euro | Eurostat (nama_10r_2gvagr) | 2021 |
|  | LIFE EXP:  Healthy life expectancy | Number of years of healthy life expected | Number | Eurostat (codes: demo_r_mlifexp & hlth_silc_17), DG Regional and Urban Policy elaboration | 3-year average 2017–2019 |
|  | HIGH_EDU:  Higher educational attainment | Population aged 25-34 with tertiary education attainment | % of population aged 25–34 | Eurostat (codes: EDAT_LFSE_04 & LFST_R_LFSD2POP) | 2021 |
|  | EMPLOY:  Employment rate (excluding agriculture) | Percentage of people aged 15-64 currently employed in all economic sectors excluding agriculture | % of population 15–64 years | Eurostat Regional Labour Force Statistics (LFS) (codes: LFST_R_LFE2EN2 & LFST_R_LFSD2POP) | 2021 |
|  | INNOV_SME: Innovative SMEs collaborating with others | SMEs with innovation cooperation activities as a percentage of the total number of SMEs. Data from the Joint European Innovation Scoreboard (EIS) and Regional Innovation Scoreboard (RIS) are presented as performance scores compared to EU performance (EU-27=100) | % of total SME | Regional Innovation Scoreboard, DG Internal Market, Industry, Entrepreneurship and SMEs – based on the Community Innovation Survey | RIS 2021 |
|  | PATENTS:  Total patent applications | Number of total patent applications per one million inhabitants | Average number 2017–2018 | DG Regional and Urban Policy, based on OECD REGPAT database | Average 2017–2018 |
|  | CREATIVE:  Core creative class employment | Population aged 15–64 classified as part of the core creative class according to the International Standard Classification of Occupations, as a percentage of the population aged 15–64 | % of population aged 15–64 | Eurostat (LFS): ad hoc extraction | Average 2019–2021 |
| Institutions | CORR_CONTRL:  The control of corruption | Pillar of the European Quality of Government Index: An aggregate of survey questions assessing corruption in the provision of public services | z-scores (the higher the better) | Quality of Government Index, Quality of Government Institute (University of Gothenburg) | 2021 |
|  | QUAL_ACCOUNT:  Quality and accountability | Pillar of the European Quality of Government Index: An aggregate of survey questions assessing the quality of public services | z-scores (the higher the better) | Quality of Government Index, Quality of Government Institute (University of Gothenburg) | 2021 |
|  | CORR_LOC_REG:  Presence of corruption in the local or regional public institutions in the country | Percentage of individuals who agree that there is corruption in local or regional public institutions | % of respondents who agree | Special Eurobarometer 470 | 2020 |

APPENDIX 3. RETI AND RDTI INDEXES CONSTRUCTION

PCA was used to weigh the individual variables and aggregate the index.

Stages:

- latent factors extraction (eigenvalues larger than 1)

- a composite factor index (CFI) for each latent factor (J) is calculated:

$$W_{KJ}=\frac{{({Factor loading}_{KJ})}^{2}}{{Explained variance}_{J}}$$

- for each indicator, the $W_{K}$ was computed as the sum of Wkj (factors with eigenvalue >1)

- calculate weights:

$$w_{K}=\frac{{\sum W}_{KJ}}{\sum W KiJ}$$

J – factors with eigenvalue >1

i – all selected indicators

- - the index was calculated as the weighted sum of the selected indicators
- $Index (RETI\&RDTI)=\sum_{i=1}^{n} w_{i}I_{ic}$

A.3.1. PCA results

3.1.1. PCA for weighting RETI

| Bartlett's sphericity test: | |
| --- | --- |
|  |  |
| Chi-square (Observed value) | 10.237 |
| Chi-square (Critical value) | 7.815 |
| DF | 3 |
| p-value (Two-tailed) | 0.017 |
| alpha | 0.05 |

Test interpretation:

H0: There is no correlation significantly different from 0 between the variables.

Ha: At least one of the correlations between the variables is significantly different from 0.

As the computed p-value is lower than the significance level alpha=0,05, one should reject the null hypothesis H0, and accept the alternative hypothesis Ha.

| Kaiser-Meyer-Olkin measure of sampling adequacy: | |
| --- | --- |
|  |  |
| GHG_GWP_2021 | 0.609 |
| GHG_GWP_11_21 | 0.606 |
| PD / 100000 inhab | 0.604 |
| KMO | 0.605 |

Eigenvalues:

|  | F1 | F2 | F3 |
| --- | --- | --- | --- |
| Eigenvalue | 1.308 | 0.972 | 0.721 |
| Variability (%) | 43.591 | 32.390 | 24.019 |
| Cumulative % | 43.591 | 75.981 | 100.000 |

| Factor loadings: |  |  |  |
| --- | --- | --- | --- |
|  |  |  |  |
|  | F1 | F2 | F3 |
| GHG_GWP_2021 | 0.537 | 0.761 | -0.363 |
| GHG_GWP_11_21 | 0.630 | -0.626 | -0.460 |
| PD / 100000 inhab | 0.789 | -0.019 | 0.614 |

A3.1.2. PCA for weighting RDTI

| Bartlett's sphericity test: |  |  |  |  |  |  |  |  |  |
| --- | --- | --- | --- | --- | --- | --- | --- | --- | --- |
|  |  |  |  |  |  |  |  |  |  |
| Chi-square (Observed value) | 559.944 |  |  |  |  |  |  |  |  |
| Chi-square (Critical value) | 32.671 |  |  |  |  |  |  |  |  |
| DF | 21 |  |  |  |  |  |  |  |  |
| p-value (Two-tailed) | < 0.0001 |  |  |  |  |  |  |  |  |
| alpha | 0.05 |  |  |  |  |  |  |  |  |
|  |  |  |  |  |  |  |  |  |  |
| Test interpretation: |  |  |  |  |  |  |  |  |  |
| H0: There is no correlation significantly different from 0 between the variables. | | | | |  |  |  |  |  |
| Ha: At least one of the correlations between the variables is significantly different from 0. | | | | | |  |  |  |  |
| As the computed p-value is lower than the significance level alpha=0.05, one should reject the  the null hypothesis H0, and accept the alternative hypothesis Ha. | | | | | | | | |  |
|  |  |  |  |  |  |  |  |  |  |
|  |  |  |  |  |  |  |  |  |  |
| Kaiser-Meyer-Olkin measure of sampling adequacy: | |  |  |  |  |  |  |  |  |
|  |  |  |  |  |  |  |  |  |  |
| Enterprises having received orders online (at least 1 %) | 0.937 |  |  |  |  |  |  |  |  |
| Enterprises with fixed broadband access. | 0.763 |  |  |  |  |  |  |  |  |
| Households with broadband access (%) | 0.700 |  |  |  |  |  |  |  |  |
| Individuals who used the internet for interaction with public authorities | 0.751 |  |  |  |  |  |  |  |  |
| Individuals buying over internet the last year | 0.757 |  |  |  |  |  |  |  |  |
| Access to high-speed broadband | 0.631 |  |  |  |  |  |  |  |  |
| Individuals with above-basic overall digital skills | 0.764 |  |  |  |  |  |  |  |  |
| KMO | 0.753 |  |  |  |  |  |  |  |  |

| Eigenvalues: |  |  |  |  |  |  |  |
| --- | --- | --- | --- | --- | --- | --- | --- |
|  |  |  |  |  |  |  |  |
|  | F1 | F2 | F3 | F4 | F5 | F6 | F7 |
| Eigenvalue | 4.237 | 1.033 | 0.558 | 0.491 | 0.439 | 0.136 | 0.107 |
| Variability (%) | 60.525 | 14.756 | 7.965 | 7.021 | 6.269 | 1.937 | 1.527 |
| Cumulative % | 60.525 | 75.281 | 83.246 | 90.267 | 96.536 | 98.473 | 100.000 |

| Factor loadings: |  |  |  |  |  |  |  |
| --- | --- | --- | --- | --- | --- | --- | --- |
|  |  |  |  |  |  |  |  |
|  | F1 | F2 | F3 | F4 | F5 | F6 | F7 |
| Enterprises having received orders online (at least 1 %) | 0.787 | -0.098 | 0.013 | -0.178 | 0.581 | 0.036 | 0.016 |
| Enterprises with fixed broadband access. | 0.749 | -0.184 | 0.603 | 0.136 | -0.073 | -0.123 | 0.048 |
| Households with broadband access (%) | 0.737 | 0.437 | -0.082 | 0.483 | 0.025 | 0.141 | 0.075 |
| Individuals who used the internet for interaction with public authorities | 0.834 | -0.323 | -0.301 | -0.156 | -0.194 | -0.038 | 0.213 |
| Individuals buying over internet the last year | 0.884 | -0.175 | -0.290 | 0.195 | -0.023 | -0.171 | -0.191 |
| Access to high-speed broadband | 0.485 | 0.810 | 0.012 | -0.296 | -0.066 | -0.128 | 0.003 |
| Individuals with above-basic overall digital skills | 0.894 | -0.085 | 0.113 | -0.242 | -0.231 | 0.229 | -0.128 |

A3.2. Weights

RETI

|  | Wkj (F1) | Wkj (F2) | Sum F1-F2 | Wk (weight) |
| --- | --- | --- | --- | --- |
| GHG_GWP_2021 | 0.220852 | 0.596323 | 0.817175 | **0.41** |
| GHG_GWP_11_21 | 0.303401 | 0.403316 | 0.706718 | **0.35** |
| PD / 100000 inhab | 0.475747 | 0.00036 | 0.476107 | **0.24** |

RDTI

|  | Wkj (F1) | Wkj (F2) | Sum F1-F2 | Wk  (weight) |
| --- | --- | --- | --- | --- |
| Access to high-speed broadband | 0.055595 | 0.634832 | 0.69042745 | **0.35** |
| Households with broadband access (%) | 0.128158 | 0.185261 | 0.31341913 | **0.16** |
| Individuals who used the Internet for interaction with public authorities | 0.164278 | 0.101118 | 0.26539544 | **0.13** |
| Individuals buying over Internet the last year | 0.184347 | 0.029734 | 0.21408163 | **0.11** |
|  |  |  |  |  |
| Individuals with above-basic overall digital skills | 0.188695 | 0.006918 | 0.19561253 | **0.10** |
| Enterprises having received orders online (at least 1 %) | 0.146354 | 0.00933 | 0.15568347 | **0.08** |
| Enterprises with fixed broadband access | 0.132573 | 0.032807 | 0.16538036 | **0.07** |

APPENDIX 4. CORRELATION ANALYSIS

- Variables: RETI; RDTI; Socio-economic indicators; Institutional indicators
- Type of correlation: Pearson
- Analised units: All EU regions (236), Selected regions (Eastern and Southern EU) (118), Eastern EU (59), Southern EU (59), Non-selected regions (Western and Northern EU) (118)
- Significance level alpha = 0.05 (5%)
- Confidence intervals (%): 95

| All EU regions | | | Selected regions | |
| --- | --- | --- | --- | --- |
| Variables | RETI(cwi) | RDTI(cw) | RETI(cwi) | RDTI(cw) |
| RETI(cwi) |  | [0.027 ; 0.276] |  | [-0.057 ; 0.298] |
| RDTI(cw) | [0.027 ; 0.276] |  | [-0.057 ; 0.298] |  |
| POP | [-0.659 ; -0.488] | [-0.013 ; 0.239] | [-0.674 ; -0.425] | [-0.080 ; 0.277] |
| GDP | [-0.065 ; 0.189] | [0.361 ; 0.561] | [-0.105 ; 0.253] | [0.198 ; 0.511] |
| LIFE_EXP | [0.255 ; 0.476] | [0.179 ; 0.411] | [0.223 ; 0.530] | [-0.156 ; 0.204] |
| HIGH_EDU | [0.011 ; 0.262] | [0.595 ; 0.736] | [-0.100 ; 0.258] | [0.552 ; 0.755] |
| EMPLOY | [-0.174 ; 0.081] | [0.384 ; 0.580] | [-0.346 ; 0.003] | [0.266 ; 0.563] |
| INNOV_SME | [0.002 ; 0.253] | [0.420 ; 0.607] | [-0.047 ; 0.306] | [0.209 ; 0.520] |
| PATENTS | [-0.153 ; 0.102] | [0.191 ; 0.422] | [-0.219 ; 0.141] | [-0.175 ; 0.185] |
| CREATIVE | [-0.092 ; 0.163] | [0.497 ; 0.665] | [-0.295 ; 0.060] | [0.330 ; 0.608] |
| CORR_LOC_REG | [-0.147 ; 0.109] | [-0.515 ; -0.303] | [0.253 ; 0.553] | [0.215 ; 0.524] |
| CORR_CONTRL | [0.104 ; 0.346] | [0.412 ; 0.601] | [-0.019 ; 0.332] | [0.285 ; 0.576] |
| QUALIT_ACCOUNT | [0.199 ; 0.428] | [0.445 ; 0.626] | [0.463 ; 0.699] | [-0.014 ; 0.336] |

- p-values (Pearson):

|  | All EU regions | | Selected regions | |
| --- | --- | --- | --- | --- |
| Variables | RETI | RDTI | RETI | RDTI |
| RETI(cwi) | **0** | **0.018** | **0** | 0.178 |
| RDTI(cw) | **0.018** | **0** | 0.178 | **0** |
| POP | **< 0.0001** | 0.078 | **< 0.0001** | 0.270 |
| GDP | 0.336 | **< 0.0001** | 0.410 | **< 0.0001** |
| LIFE_EXP | **< 0.0001** | **< 0.0001** | **< 0.0001** | 0.789 |
| HIGH_EDU | **0.033** | **< 0.0001** | 0.377 | **< 0.0001** |
| EMPLOY | 0.473 | **< 0.0001** | 0.054 | **< 0.0001** |
| INNOV_SME | **0.046** | **< 0.0001** | 0.147 | **< 0.0001** |
| PATENTS | 0.696 | **< 0.0001** | 0.662 | 0.958 |
| CREATIVE | 0.585 | **< 0.0001** | 0.188 | **< 0.0001** |
| CORR_LOC_REG | 0.768 | **< 0.0001** | **< 0.0001** | **< 0.0001** |
| CORR_CONTRL | **0.000** | **< 0.0001** | 0.079 | **< 0.0001** |
| QUALIT_ACCOUNT | **< 0.0001** | **< 0.0001** | **< 0.0001** | 0.070 |
|  |  |  |  |  |

- Correlation matrix:

**All EU regions**

**Selected regions**

APPENDIX 5. REGRESSION ANALYSIS

**A5.1. All EU regions (sample: 236)**

- - 1. ***Dependent variable: RETI (All EU regions)***

| Confidence interval (%): 95 | |  |
| --- | --- | --- |
| Tolerance: 0.0001 |  |  |
| Validation: Random |  |  |
| Number of observations for the validation: 1 | | |
| Model selection: Best model / Adjusted R² | | |
| Min variables: 2 / Max variables: 5 | | |

|  | Tolerance | VIF |
| --- | --- | --- |
| POP | 0.793 | 1.261 |
| GDP | 0.130 | 7.670 |
| LIFE_EXP | 0.486 | 2.059 |
| HIGH_EDU | 0.352 | 2.844 |
| INNOV_SME | 0.105 | 9.494 |
| PATENTS | 0.512 | 1.955 |
| CREATIVE | 0.209 | 4.794 |
| CORR_CONTRL | 0.097 | 9.283 |
| QUALIT_ACCOUNT | 0.158 | 6.329 |
| CORR_LOC_REG | 0.309 | 3.233 |

Goodness of fit statistics RETI:

| Statistic | Training set | Validation set |
| --- | --- | --- |
| Observations | 235.000 | 1.000 |
| Sum of weights | 235.000 | 1.000 |
| DF | 229.000 | -5.000 |
| R² | 0.575 |  |
| Adjusted R² | 0.566 |  |
| MSE | 0.011 |  |
| RMSE | 0.104 |  |
| MAPE | 12.771 | 0.000 |
| DW | 1.368 |  |
| Cp | 5.612 |  |
| AIC | -1059.766 |  |
| SBC | -1039.008 |  |
| PC | 0.447 |  |
| Press | 2.668 |  |
| Q² | 0.539 | 0.000 |

| Analysis of variance RETI: | | | |  |  |
| --- | --- | --- | --- | --- | --- |
|  |  |  |  |  |  |
| Source | DF | Sum of squares | Mean squares | F | Pr > F |
| Model | 5 | 3.326 | 0.665 | 61.997 | **< 0.0001** |
| Error | 229 | 2.457 | 0.011 |  |  |
| Corrected Total | 234 | 5.783 |  |  |  |
| *Computed against model Y=Mean(Y)* | | | |  |  |

| Model parameters RETI: | | |  |  |  |  |
| --- | --- | --- | --- | --- | --- | --- |
|  |  |  |  |  |  |  |
| Source | Value | Standard error | t | Pr > \|t\| | Lower bound (95%) | Upper bound (95%) |
| Intercept | 0.449 | 0.043 | 10.458 | **< 0.0001** | 0.365 | 0.534 |
| POP | -0.759 | 0.052 | -14.483 | **< 0.0001** | -0.862 | -0.656 |
| GDP | 0.000 | 0.000 |  |  |  |  |
| LIFE_EXP | 0.233 | 0.033 | 7.139 | **< 0.0001** | 0.169 | 0.297 |
| HIGH_EDU | 0.092 | 0.047 | 1.945 | 0.053 | -0.001 | 0.185 |
| INNOV_SME | 0.000 | 0.000 |  |  |  |  |
| PATENTS | 0.000 | 0.000 |  |  |  |  |
| CREATIVE | 0.000 | 0.000 |  |  |  |  |
| CORR_CONTRL | 0.000 | 0.000 |  |  |  |  |
| QUALIT_ACCOUNT | 0.120 | 0.050 | 2.399 | **0.017** | 0.021 | 0.218 |
| CORR_LOC_REG | 0.145 | 0.036 | 4.007 | **< 0.0001** | 0.074 | 0.216 |

Equation of the model RETI:

RETI = 0,45-0,76*POP+0,23*LIFE_EXP+0,09*HIGH_EDU+0,12*QUALIT_ACCOUNT+0,14*CORR_LOC_REG

Standardized coefficients RETI

| Source | Value | Standard error | t | Pr > \|t\| | Lower bound (95%) | Upper bound (95%) |
| --- | --- | --- | --- | --- | --- | --- |
| POP | -0.644 | 0.044 | -14.483 | **< 0.0001** | -0.732 | -0.556 |
| GDP | 0.000 | 0.000 |  |  |  |  |
| LIFE_EXP | 0.379 | 0.053 | 7.139 | **< 0.0001** | 0.274 | 0.483 |
| HIGH_EDU | 0.096 | 0.050 | 1.945 | 0.053 | -0.001 | 0.194 |
| INNOV_SME | 0.000 | 0.000 |  |  |  |  |
| PATENTS | 0.000 | 0.000 |  |  |  |  |
| CREATIVE | 0.000 | 0.000 |  |  |  |  |
| CORR_CONTRL | 0.000 | 0.000 |  |  |  |  |
| QUALIT_ACCOUNT | 0.163 | 0.068 | 2.399 | **0.017** | 0.029 | 0.297 |
| CORR_LOC_REG | 0.220 | 0.055 | 4.007 | **< 0.0001** | 0.112 | 0.328 |

*Interpretation RETI*:

Using the Best model variables selection method, 5 variables have been retained in the model.

Given the R^2^, the 5 explanatory variables explain 58% of the variability of the dependent variable RETI.

Considering the p-value of the F statistic computed in the ANOVA table and the significance level of 5%, the information provided by the explanatory variables is significantly better than that offered by a basic mean.

Based on the Type III sum of squares, the following variables present significant information to explain the variability of the dependent variable RETI(cwi): LIFE_EXP, CORR_LOC_REG, and QUALIT_ACCOUNT.

- - 1. ***Dependent variable: RDTI (All EU regions)***

| Confidence interval (%): 95 | | | | | | |  | |  |  |  |  |  |
| --- | --- | --- | --- | --- | --- | --- | --- | --- | --- | --- | --- | --- | --- |
| Tolerance: 0.0001 | | | | |  | |  | |  |  |  |  |  |
| Validation: Random | | | | |  | |  | |  |  |  |  |  |
| Number of observations for the validation: 1 | | | | | | | | |  |  |  |  |  |
| Model selection: Best model / Adjusted R² | | | | | | | | |  |  |  |  |  |
| Min variables: 2 / Max variables: 5 | | | | | | | | |  |  |  |  |  |
| Multicolinearity statistics: | | | | | | | | |  |  |  |  |  |
|  | | Tolerance | | VIF | | | |  |  |  |  |  |  |
| POP | | 0.783 | | 1.277 | | | |  |  |  |  |  |  |
| GDP | | 0.112 | | 8.926 | | | |  |  |  |  |  |  |
| LIFE_EXP | | 0.409 | | 2.448 | | | |  |  |  |  |  |  |
| HIGH_EDU | | 0.366 | | 2.735 | | | |  |  |  |  |  |  |
| EMPLOY | | 0.266 | | 3.753 | | | |  |  |  |  |  |  |
| INNOV_SME | | 0.101 | | 9.856 | | | |  |  |  |  |  |  |
| PATENTS | | 0.497 | | 2.012 | | | |  |  |  |  |  |  |
| CREATIVE | | 0.208 | | 4.806 | | | |  |  |  |  |  |  |
| QUALIT_ACCOUNT | | 0.148 | | 6.755 | | | |  |  |  |  |  |  |
| CORR_CONTRL | | 0.121 | | 8.251 | | | |  |  |  |  |  |  |
|  | | | | | | | | | |  |  |  |  |
| Goodness of fit statistics RDTI: | | | | | |  |  |  |  |  |  |  |  |
|  |  | |  | | |  |  |  |  |  |  |  |  |
| Statistic | Training set | | Validation set | | |  |  |  |  |  |  |  |  |
| Observations | 235.000 | | 1.000 | | |  |  |  |  |  |  |  |  |
| Sum of weights | 235.000 | | 1.000 | | |  |  |  |  |  |  |  |  |
| DF | 229.000 | | -5.000 | | |  |  |  |  |  |  |  |  |
| R² | 0.555 | |  | | |  |  |  |  |  |  |  |  |
| Adjusted R² | 0.545 | |  | | |  |  |  |  |  |  |  |  |
| MSE | 0.024 | |  | | |  |  |  |  |  |  |  |  |
| RMSE | 0.155 | |  | | |  |  |  |  |  |  |  |  |
| MAPE | 43.606 | | 0.000 | | |  |  |  |  |  |  |  |  |
| DW | 0.980 | |  | | |  |  |  |  |  |  |  |  |
| Cp | 4.544 | |  | | |  |  |  |  |  |  |  |  |
| AIC | -869.314 | |  | | |  |  |  |  |  |  |  |  |
| SBC | -848.557 | |  | | |  |  |  |  |  |  |  |  |
| PC | 0.469 | |  | | |  |  |  |  |  |  |  |  |
| Press | 5.865 | |  | | |  |  |  |  |  |  |  |  |
| Q² | 0.527 | | 0.000 | | |  |  |  |  |  |  |  |  |

| Analysis of variance (RDTI(cw)): | | | |  |  |
| --- | --- | --- | --- | --- | --- |
|  |  |  |  |  |  |
| Source | DF | Sum of squares | Mean squares | F | Pr > F |
| Model | 5 | 6.885 | 1.377 | 57.070 | **< 0.0001** |
| Error | 229 | 5.525 | 0.024 |  |  |
| Corrected Total | 234 | 12.410 |  |  |  |
| *Computed against model Y=Mean(Y)* | | | |  |  |

| Type III Sum of Squares analysis RDTI: | | | | |  |
| --- | --- | --- | --- | --- | --- |
|  |  |  |  |  |  |
| Source | DF | Sum of squares | Mean squares | F | Pr > F |
| POP | 0 | 0.000 |  |  |  |
| GDP | 1 | 0.309 | 0.309 | 12.806 | **0.000** |
| LIFE_EXP | 0 | 0.000 |  |  |  |
| HIGH_EDU | 1 | 2.328 | 2.328 | 96.504 | **< 0.0001** |
| EMPLOY | 1 | 0.171 | 0.171 | 7.077 | **0.008** |
| INNOV_SME | 1 | 0.443 | 0.443 | 18.378 | **< 0.0001** |
| PATENTS | 0 | 0.000 |  |  |  |
| CREATIVE | 0 | 0.000 |  |  |  |
| QUALIT_ACCOUNT | 1 | 0.062 | 0.062 | 2.581 | 0.110 |
| CORR_CONTRL | 0 | 0.000 |  |  |  |

Model parameters (RDTI(cw)):

| Source | Value | Standard error | t | Pr > \|t\| | Lower bound (95%) | Upper bound (95%) |
| --- | --- | --- | --- | --- | --- | --- |
| Intercept | -0.157 | 0.043 | -3.647 | **0.000** | -0.242 | -0.072 |
| POP | 0.000 | 0.000 |  |  |  |  |
| GDP | -0.549 | 0.154 | -3.579 | **0.000** | -0.852 | -0.247 |
| LIFE_EXP | 0.000 | 0.000 |  |  |  |  |
| HIGH_EDU | 0.757 | 0.077 | 9.824 | **< 0.0001** | 0.605 | 0.908 |
| EMPLOY | 0.200 | 0.075 | 2.660 | **0.008** | 0.052 | 0.348 |
| INNOV_SME | 0.627 | 0.146 | 4.287 | **< 0.0001** | 0.339 | 0.915 |
| PATENTS | 0.000 | 0.000 |  |  |  |  |
| CREATIVE | 0.000 | 0.000 |  |  |  |  |
| QUALIT_ACCOUNT | 0.117 | 0.073 | 1.606 | 0.110 | -0.026 | 0.260 |
| CORR_CONTRL | 0.000 | 0.000 |  |  |  |  |

Equation of the model RDTI:

RDTI = -0,16-0,55*GDP+0,76*HIGH_EDU+0,20*EMPLOY+0,63*INNOV_SME+0,12*QUALIT_ACCOUNT

Standardized coefficients RDTI

| Source | Value | Standard error | t | Pr > \|t\| | Lower bound (95%) | Upper bound (95%) |
| --- | --- | --- | --- | --- | --- | --- |
| POP | 0.000 | 0.000 |  |  |  |  |
| GDP | -0.369 | 0.103 | -3.579 | **0.000** | -0.573 | -0.166 |
| LIFE_EXP | 0.000 | 0.000 |  |  |  |  |
| HIGH_EDU | 0.542 | 0.055 | 9.824 | **< 0.0001** | 0.433 | 0.650 |
| EMPLOY | 0.176 | 0.066 | 2.660 | **0.008** | 0.046 | 0.307 |
| INNOV_SME | 0.424 | 0.099 | 4.287 | **< 0.0001** | 0.229 | 0.618 |
| PATENTS | 0.000 | 0.000 |  |  |  |  |
| CREATIVE | 0.000 | 0.000 |  |  |  |  |
| QUALIT_ACCOUNT | 0.109 | 0.068 | 1.606 | 0.110 | -0.025 | 0.242 |
| CORR_CONTRL | 0.000 | 0.000 |  |  |  |  |

*Interpretation RDTI:*

Using the Best model variables selection method, 5 variables have been retained in the model.

Given the R^2^, 55% of the variability of the dependent variable RDTI is explained by the 5 explanatory variables.

Considering the p-value of the F statistic computed in the ANOVA table and the significance level of 5%, the information provided by the explanatory variables is significantly better than that offered by a basic mean.

Based on the Type III sum of squares, the following variables bring significant information to explain the variability of the dependent variable RDTI: HIGH_EDU, INNOV_SME, and EMPLOY.

**A5.2. Selected regions (sample: 236)**

|  | Tolerance | VIF |
| --- | --- | --- |
| POP | 0.684 | 1.462 |
| GDP | 0.093 | 9.721 |
| LIFE_EXP | 0.163 | 6.146 |
| HIGH_EDU | 0.256 | 3.900 |
| EMPLOY | 0.240 | 4.159 |
| INNOV_SME | 0.090 | 9.099 |
| PATENTS | 0.299 | 3.345 |
| CREATIVE | 0.193 | 5.178 |
| CORR_CONTRL | 0.288 | 3.469 |
| QUALIT_ACCOUNT | 0.233 | 4.288 |
| CORR_LOC_REG | 0.453 | 2.208 |

***5.2.1. Dependent variable: RETI (Selected regions)***

| Confidence interval (%): 95 | |  |
| --- | --- | --- |
| Tolerance: 0,0001 |  |  |
| Validation: Random |  |  |
| Number of observations for the validation: 1 | | |
| Model selection: Best model / Adjusted R² | | |
| Min variables: 2 / Max variables: 5 | | |

| Goodness of fit statistics RETI: | | |
| --- | --- | --- |
|  |  |  |
| Statistic | Training set | Validation set |
| Observations | 118.000 | 1.000 |
| Sum of weights | 118.000 | 1.000 |
| DF | 112.000 | -5.000 |
| R² | 0.703 |  |
| Adjusted R² | 0.690 |  |
| MSE | 0.008 |  |
| RMSE | 0.091 |  |
| MAPE | 10.983 | 0.000 |
| DW | 1.877 |  |
| Cp | 7.728 |  |
| AIC | -559.092 |  |
| SBC | -542.468 |  |
| PC | 0.328 |  |
| Press | 1.048 |  |
| Q² | 0.667 | 0.000 |

| Analysis of variance RETI | |  |  |  |  |
| --- | --- | --- | --- | --- | --- |
|  |  |  |  |  |  |
| Source | DF | Sum of squares | Mean squares | F | Pr > F |
| Model | 5 | 2.213 | 0.443 | 53.127 | **< 0,0001** |
| Error | 112 | 0.933 | 0.008 |  |  |
| Corrected Total | 117 | 3.147 |  |  |  |
| *Computed against model Y=Mean(Y)* | | |  |  |  |

| Type III Sum of Squares analysis RETI: | | |  |  |  |
| --- | --- | --- | --- | --- | --- |
|  |  |  |  |  |  |
| Source | DF | Sum of squares | Mean squares | F | Pr > F |
| POP | 1 | 0.939 | 0.939 | 112.678 | **< 0.0001** |
| GDP | 0 | 0.000 |  |  |  |
| LIFE_EXP | 0 | 0.000 |  |  |  |
| HIGH_EDU | 0 | 0.000 |  |  |  |
| EMPLOY | 1 | 0.072 | 0.072 | 8.583 | **0.004** |
| INNOV_SME | 1 | 0.107 | 0.107 | 12.889 | **0.000** |
| PATENTS | 0 | 0.000 |  |  |  |
| CREATIVE | 0 | 0.000 |  |  |  |
| CORR_CONTRL | 0 | 0.000 |  |  |  |
| QUALIT_ACCOUNT | 1 | 0.088 | 0.088 | 10.571 | **0.002** |
| CORR_LOC_REG | 1 | 0.308 | 0.308 | 36.923 | **< 0.0001** |

| Model parameters RETI: | |  |  |  |  |  |
| --- | --- | --- | --- | --- | --- | --- |
|  |  |  |  |  |  |  |
| Source | Value | Standard error | t | Pr > \|t\| | Lower bound (95%) | Upper bound (95%) |
| Intercept | 0.335 | 0.070 | 4.758 | **< 0.0001** | 0.195 | 0.474 |
| POP | -0.701 | 0.066 | -10.615 | **< 0.0001** | -0.832 | -0.570 |
| GDP | 0.000 | 0.000 |  |  |  |  |
| LIFE_EXP | 0.000 | 0.000 |  |  |  |  |
| HIGH_EDU | 0.000 | 0.000 |  |  |  |  |
| EMPLOY | -0.152 | 0.052 | -2.930 | **0.004** | -0.255 | -0.049 |
| INNOV_SME | 0.339 | 0.095 | 3.590 | **0.000** | 0.152 | 0.527 |
| PATENTS | 0.000 | 0.000 |  |  |  |  |
| CREATIVE | 0.000 | 0.000 |  |  |  |  |
| CORR_CONTRL | 0.000 | 0.000 |  |  |  |  |
| QUALIT_ACCOUNT | 0.194 | 0.060 | 3.251 | **0.002** | 0.076 | 0.313 |
| CORR_LOC_REG | 0.465 | 0.076 | 6.076 | **< 0.0001** | 0.313 | 0.616 |

Equation of the model RETI:

RETI= 0,33-0,70*POP-0,15*EMPLOY+0,34*INNOV_SME+0,19*QUALIT_ACCOUNT+0,46*CORR_LOC_REG

| Standardized coefficients RETI: | | |  |  |  |  |
| --- | --- | --- | --- | --- | --- | --- |
|  |  |  |  |  |  |  |
| Source | Value | Standard error | t | Pr > \|t\| | Lower bound (95%) | Upper bound (95%) |
| POP | -0.599 | 0.056 | -10.615 | **< 0.0001** | -0.711 | -0.487 |
| GDP | 0.000 | 0.000 |  |  |  |  |
| LIFE_EXP | 0.000 | 0.000 |  |  |  |  |
| HIGH_EDU | 0.000 | 0.000 |  |  |  |  |
| EMPLOY | -0.183 | 0.062 | -2.930 | **0.004** | -0.307 | -0.059 |
| INNOV_SME | 0.231 | 0.064 | 3.590 | **0.000** | 0.104 | 0.359 |
| PATENTS | 0.000 | 0.000 |  |  |  |  |
| CREATIVE | 0.000 | 0.000 |  |  |  |  |
| CORR_CONTRL | 0.000 | 0.000 |  |  |  |  |
| QUALIT_ACCOUNT | 0.198 | 0.061 | 3.251 | **0.002** | 0.078 | 0.319 |
| CORR_LOC_REG | 0.382 | 0.063 | 6.076 | **< 0.0001** | 0.257 | 0.506 |

*Interpretation RETI:*

Using the Best model variables selection method, 5 variables have been retained in the model.

Given the R2, 70% of the variability of the dependent variable RETI is explained by the 5 explanatory variables.

Considering the p-value of the F statistic computed in the ANOVA table and the significance level of 5%, the information provided by the explanatory variables is significantly better than that offered by a basic mean.

Based on the Type III sum of squares, the following variables bring significant information to explain the variability of the dependent variable RETI: CORR_LOC_REG, INNOV_SME, and QUALIT_ACCOUNT.

- - 1. ***Dependent variable: RDTI (Selected regions)***

|  |  | |  |  |  |
| --- | --- | --- | --- | --- | --- |
| Goodness of fit statistics (RDTI(cw)): | | | | | |
|  | |  | | |  |
| Statistic | | Training set | | | Validation set |
| Observations | | 118.000 | | | 1.000 |
| Sum of weights | | 118.000 | | | 1.000 |
| DF | | 112.000 | | | -5.000 |
| R² | | 0.611 | | |  |
| Adjusted R² | | 0.594 | | |  |
| MSE | | 0.017 | | |  |
| RMSE | | 0.132 | | |  |
| MAPE | | 59.621 | | | 0.000 |
| DW | | 1.048 | | |  |
| Cp | | 21.967 | | |  |
| AIC | | -472.112 | | |  |
| SBC | | -455.487 | | |  |
| PC | | 0.431 | | |  |
| Press | | 2.165 | | |  |
| Q² | | 0.568 | | | 0.000 |

| Analysis of variance RDTI: | | | |  | |  | |  | |  | |
| --- | --- | --- | --- | --- | --- | --- | --- | --- | --- | --- | --- |
|  | | |  |  | |  | |  | |  | |
| Source | | | DF | Sum of squares | | Mean squares | | F | | Pr > F | |
| Model | | | 5 | 3.065 | | 0.613 | | 35.199 | | **< 0.0001** | |
| Error | | | 112 | 1.950 | | 0.017 | |  | |  | |
| Corrected Total | | | 117 | 5.015 | |  | |  | |  | |
| *Computed against model Y=Mean(Y)* | | | | | |  | |  | |  | |
| Type III Sum of Squares analysis RDTI: | | | | |  | |  | |  | |  |
|  |  |  | | |  | |  | |  | |  |
| Source | DF | Sum of squares | | | Mean squares | | F | | Pr > F | |  |
| POP | 0 | 0.000 | | |  | |  | |  | |  |
| GDP | 1 | 0.315 | | | 0.315 | | 18.082 | | **< 0.0001** | |  |
| LIFE_EXP | 1 | 0.539 | | | 0.539 | | 30.979 | | **< 0.0001** | |  |
| HIGH_EDU | 1 | 1.554 | | | 1.554 | | 89.255 | | **< 0.0001** | |  |
| EMPLOY | 0 | 0.000 | | |  | |  | |  | |  |
| INNOV_SME | 1 | 0.685 | | | 0.685 | | 39.311 | | **< 0.0001** | |  |
| PATENTS | 0 | 0.000 | | |  | |  | |  | |  |
| CREATIVE | 0 | 0.000 | | |  | |  | |  | |  |
| CORR_CONTRL | 0 | 0.000 | | |  | |  | |  | |  |
| QUALIT_ACCOUNT | 0 | 0.000 | | |  | |  | |  | |  |
| CORR_LOC_REG | 1 | 0.266 | | | 0.266 | | 15.258 | | **0.000** | |  |

| Model parameters RDTI: | |  |  |  |  |  |
| --- | --- | --- | --- | --- | --- | --- |
|  |  |  |  |  |  |  |
| Source | Value | Standard error | t | Pr > \|t\| | Lower bound (95%) | Upper bound (95%) |
| Intercept | -0.294 | 0.086 | -3.430 | **0.001** | -0.464 | -0.124 |
| POP | 0.000 | 0.000 |  |  |  |  |
| GDP | -0.991 | 0.233 | -4.252 | **< 0.0001** | -1.453 | -0.529 |
| LIFE_EXP | -0.379 | 0.068 | -5.566 | **< 0.0001** | -0.514 | -0.244 |
| HIGH_EDU | 0.796 | 0.084 | 9.447 | **< 0.0001** | 0.629 | 0.963 |
| EMPLOY | 0.000 | 0.000 |  |  |  |  |
| INNOV_SME | 1.788 | 0.285 | 6.270 | **< 0.0001** | 1.223 | 2.353 |
| PATENTS | 0.000 | 0.000 |  |  |  |  |
| CREATIVE | 0.000 | 0.000 |  |  |  |  |
| CORR_CONTRL | 0.000 | 0.000 |  |  |  |  |
| QUALIT_ACCOUNT | 0.000 | 0.000 |  |  |  |  |
| CORR_LOC_REG | 0.449 | 0.115 | 3.906 | **0.000** | 0.221 | 0.676 |

| Equation of the model RDTI: | | | | | | |
| --- | --- | --- | --- | --- | --- | --- |
| RDTI = -0,29-0,99*GDP-0,37*LIFE_EXP+0,79*HIGH_EDU+1,79*INNOV_SME+0,45*CORR_LOC_REG | | | | | | |
| Standardized coefficients RDTI: | | |  |  |  |  |
|  |  |  |  |  |  |  |
| Source | Value | Standard error | t | Pr > \|t\| | Lower bound (95%) | Upper bound (95%) |
| POP | 0,000 | 0,000 |  |  |  |  |
| GDP | -0,580 | 0,136 | -4,252 | **< 0,0001** | -0,851 | -0,310 |
| LIFE_EXP | -0,563 | 0,101 | -5,566 | **< 0,0001** | -0,764 | -0,363 |
| HIGH_EDU | 0,651 | 0,069 | 9,447 | **< 0,0001** | 0,514 | 0,787 |
| EMPLOY | 0,000 | 0,000 |  |  |  |  |
| INNOV_SME | 0,965 | 0,154 | 6,270 | **< 0,0001** | 0,660 | 1,270 |
| PATENTS | 0,000 | 0,000 |  |  |  |  |
| CREATIVE | 0,000 | 0,000 |  |  |  |  |
| CORR_CONTRL | 0,000 | 0,000 |  |  |  |  |
| QUALIT_ACCOUNT | 0,000 | 0,000 |  |  |  |  |
| CORR_LOC_REG | 0,292 | 0,075 | 3,906 | **0,000** | 0,144 | 0,440 |

*Interpretation RDTI:*

Using the Best model variables selection method, 5 variables have been retained in the model.

Given the R^2^, the 5 explanatory variables explain 61% of the variability of the dependent variable RDTI.

Considering the p-value of the F statistic computed in the ANOVA table and the significance level of 5%, the information provided by the explanatory variables is significantly better than that offered by a basic mean.

Based on the Type III sum of squares, the following variables bring significant information to explain the variability of the dependent variable RDTI(cw): INNOV_SME, HIGH_EDU, and CORR_LOC_REG.
